# Galectin-9 is required for endometrial regenerative cells to induce long-term cardiac allograft survival in mice

**DOI:** 10.1186/s13287-020-01985-0

**Published:** 2020-11-05

**Authors:** Yiming Zhao, Xiang Li, Dingding Yu, Yonghao Hu, Wang Jin, Yafei Qin, Dejun Kong, Hongda Wang, Guangming Li, Alessandro Alessandrini, Hao Wang

**Affiliations:** 1 Department of General Surgery, Tianjin Medical University General Hospital, 154 Anshan Road, Heping District, Tianjin, 300052 China; 2 Tianjin General Surgery Institute, Tianjin Medical University General Hospital, Tianjin, China; 3 Department of Surgery, Center for Transplantation Sciences, Massachusetts General Hospital and Harvard Medical School, Boston, MA USA

**Keywords:** Endometrial regenerative cells, Cardiac allograft rejection, Galectin-9, Immunoregulation, Rapamycin

## Abstract

**Background:**

Endometrial regenerative cells (ERCs), a novel type of mesenchymal-like stem cells, were identified as an attractive candidate for immunoregulation and induction of cardiac allograft tolerance. However, the underlying mechanisms of ERCs in immune regulation still remain largely unclear. The present study is designed to determine whether the expression of Galectin-9 (Gal-9), a soluble tandem-repeat member of the galectin family, is crucial for ERC-based immunomodulation.

**Methods:**

In this study, we measured Gal-9 expression on ERCs and then co-cultured Gal-9-ERCs, ERCs, and ERCs+lactose (Gal-9 blocker) with activated C57BL/6-derived splenocytes. Furthermore, we performed mouse heart transplantation between BALB/c (H-2^d^) donor and C57BL/6 (H-2^b^) recipient. ERCs were administrated 24 h after the surgery, either alone or in combination with rapamycin.

**Results:**

Our data demonstrate that ERCs express Gal-9, and this expression is increased by IFN-γ stimulation in a dose-dependent manner. Moreover, both in vitro and in vivo results show that Gal-9-ERC-mediated therapy significantly suppressed Th1 and Th17 cell response, inhibited CD8^+^ T cell proliferation, abrogated B cell activation, decreased donor-specific antibody production, and enhanced the Treg population. The therapeutic effect of ERCs was further verified by their roles in prolonging cardiac allograft survival and alleviating graft pathological changes.

**Conclusions:**

Taken together, these data indicate that Gal-9 is required for ERC-mediated immunomodulation and prevention of allograft rejection.

## Background

Organ transplantation is an effective substitute and supportive treatment for tumor, irreversible organ damage, and end-stage organ failure [1, 2]. The diversity of individual major histocompatibility complex (MHC) greatly restricts the survival of organ transplants [3]. Thankfully, in recent years, with the application of immunosuppressants, short-term allograft survival rate has greatly improved, whereas cardiac allograft vasculopathy (CAV) caused by chronic rejection still affects the long-term survival of the transplanted organs [4].

Meanwhile, for the recipients, the side effects of long-term and high-dose use of immunosuppressive agents should not be underestimated. Long-period immune response suppression results in increased susceptibility to infection, malignancy, and bone marrow suppression [5]. Furthermore, large doses of immunosuppresants could also cause a series of metabolic diseases, such as diabetes, hypertension, and hypercholesterolemia [6]. Therefore, it is of great significance to find a novel immunomodulatory therapy to supplement the current clinical treatment strategies.

Mesenchymal stem cells (MSCs) are a type of pluripotent stem cells that have the potential for self-renewal, multi-directional differentiation, and immune regulation. The lacking expression of MHC I, MHC II, and costimulatory molecules results in its low immunogenicity and satisfactory acceptance by the recipients [7]. Additionally, MSCs have the ability to mobilize, undergo chemotaxis, and are immunoregulatory, which makes them great candidates in mediating immune tolerance in somatic organ transplantation [8]. However, there are also some disadvantages in the use of MSCs as therapeutic agents. Acquisition of adult MSCs is a very invasive process, and it is difficult to obtain large quantities of cells. Furthermore, the proliferation and expansion capabilities of MSCs are also limited, and under certain conditions, MSCs could even promote tumor expansion and metastasis [9]. Thus, there is a need in seeking a novel source of stem cells with a better risk-to-benefit profile.

Endometrial regenerative cells (ERCs), which are mesenchymal-like stem cells, collected from menstrual blood, were identified as a new candidate for immune regulation [10]. When compared with traditional MSCs, ERCs have certain advantages, such as abundant resource, non-invasive collection methods, reusing human waste, and easy to large-scale expansion capability. In addition, ERCs were also reported with the ability in modulating immune response and lack of immunogenicity [11]. Previous studies from our research group and others have reported that ERC-mediated stem cell therapy could modulate the immune response in the treatment of a series of diseases, such as stroke, myocardial infarction, ischemia reperfusion injury, inflammatory bowel disease, and multiple organ dysfunction syndrome [12]. Recently, we have also discovered that ERCs could secrete SDF-1, the ligand for C-X-C chemokine receptor type 4 (CXCR-4), which could promote ERC migration, chemotaxis, and aggregation at the injury site and mediate allograft tolerance. However, the specific immunomodulatory mechanisms concerned are still undefined [13].

Galectin-9 (Gal-9), a soluble tandem-repeat member of the galectin family, specifically binds to β-galactosides [14], while T cell immunoglobulin mucin-3 (Tim-3), the ligand for Gal-9, expresses on the surface of CD8^+^ T, T helper type 1 (Th1), and a portion of Th17 cells [15]. Blocking Tim-3 signal by application of anti-TIM-3 mAb was previously verified in accelerating cardiac allograft rejection [16]. Further data also indicated that infusion of Tim-3-Ig could aggravate acute graft-vs-host disease (aGVHD) [17]. Through Gal-9-Tim-3 interaction, Gal-9 could negatively regulate Th1 immunity, suppress the generation of Th17 cells, and inhibit CD8^+^ T cell response [18, 19]. Besides, Gal-9 was verified in regulating B cell signaling transduction by binding with IgM-BCR. When adopting exogenous recombinant Gal-9, it could nearly completely abolish BCR signaling transduction [20, 21]. Further experiments also suggested that exogenous Gal-9 was required for induced regulatory T cell (iTreg) differentiation and maintenance [22].

Given the promising immunomodulatory capability of Gal-9, exploring their effects in ERCs was proposed. Till now, there is still no relevant study reported focusing on evaluating Gal-9 role in mediating ERC immunomodulatory effects. And only few researches documented that Gal-9 was presented as a predictor for MSC modulatory effect in vitro [23]. Meanwhile, multiple studies have revealed that IFN-γ-activated stem cells exhibit a stronger differentiation and proliferation ability, in addition to vital phenotype changes, resulting in their extraordinary immune regulation capability [24, 25].

Inspired by the previous investigations, the present study is designed to explore whether Gal-9 is expressed on ERCs, and whether Gal-9-mediated stem cell therapy could assist in alleviating allo-immune response, therefore promoting the long-term survival of cardiac allografts.

## Materials and methods

### Animals

Male C57BL/6 (B6, H-2^b^) and BALB/c (H-2^d^) mice weighing 22–25 g were purchased from the China Food and Drug Inspection Institute (Beijing, China). All the mice were housed in a constant temperature room with a 12-h dark/12-h light cycle and supplied with free access to autoclaved food and fresh water. All the experiments were conducted in conformity with the standard protocols approved by the Animal Care and Use Committee of Tianjin Medical University (Tianjin, China), according to the Chinese Council on Animal Care guidelines.

### ERC harvest, isolation, and phenotype identification

ERC preparation methods were followed by the protocols described previously [13]. In brief, menstrual blood was collected by using a sterilized menstrual cup on the first day of the menstrual cycle from a healthy female volunteer (20–40 years old). After being filtered and suspended in MEM medium, the harvested suspension was then added into Ficoll lymphocyte separation solution and centrifuged to obtain the monocyte layer cells. The obtained cells were resuspended in α-MEM complete medium (Hyclone, USA), which is supplemented with 10% fetal bovine serum (Hyclone, USA) and 1% penicillin/streptomycin (Solarbio, Beijing, China), and then seeded in 6-well plates. The medium was changed every 3 days to remove the non-adherent cells. After culturing for 2 weeks, the cells appeared with a spindle-shape morphology and were distributed equally. Different generations of ERCs were photographed for record, and the uniform third-generation ERCs were collected to detect the surface marker expression (CD29, CD45, CD90, and CD105), to further confirm their stem cell characteristics.

### Identification of Gal-9 expression on ERCs

ERC cell homogenates (2 × 10^5^) and supernatant from the 2nd to 5th generations were collected to detect the expression of Gal-9. Meanwhile, different concentrations of IFN-γ (0–20 ng/ml, PeproTech, Rocky Hill, USA) were selected to pre-stimulate ERC cells for 72 h, and then Gal-9 expression changes in ERC cell lysate and supernatant were measured by ELISA Kit. Based on the results that IFN-γ pre-stimulation (20 ng/ml, 72 h) could significantly increase Gal-9 expression in ERC lysates, we set up as the control group (ERCs without pre-treatment) and ERC + IFN-γ group (IFN-γ pre-stimulation, 20 ng/ml, 72 h). Flow cytometry, western blot, and RT-PCR were performed respectively to qualitatively and quantitatively verify the Gal-9 expression difference. Furthermore, in order to more intuitively observe the changes of Gal-9 expression on ERCs, we adopted immunofluorescence staining to directly and clearly define Gal-9 expression changes. Given the above results, the follow-up ERC treatment group with Gal-9 high expression was all pre-stimulated with IFN-γ for 72 h.

### Co-culture of ERCs with activated B6-derived splenocytes

In order to determine the modulation effect of ERC-derived Gal-9 on the proportion and function of spleen cells, then the in vitro co-culture experiment was performed. Briefly speaking, splenocytes (5 × 10^5^) obtained from C57BL/6 mice were inoculated into a 48-well plate and activated by the following stimulants: For Th1, Th17, and regulatory T cells (Tregs), the stimulators were IL-2 + anti-mouse CD3 (100 ng/ml) and CD28 (200 ng/ml) antibodies (ebioscience Inc., USA), and for B cells, the stimulator was lipopolysaccharide (10 ng/ml, R&D, USA). However, there were no stimulants added into the control group. Meanwhile, ERCs, IFN-γ-pre-stimulated ERCs (20 ng/ml, 72 h), and lactose antagonistic (10.8 mg/ml, Sigma-Aldrich) ERCs (2.5 × 10^4^) were added to each group respectively. After 96 h of co-cultivation, the supernatant was obtained to measure the IgG and IgM secretion, and the cell substrate was collected for RT-PCR or flow cytometry analysis to detect cytokine expression and proportion changes in the splenocytes. It is worth noting that lactose was reported sharing the similar epitope with Gal-9, which was shown that it could competitively antagonize Gal-9 binding ligand and block its immunomodulatory effects [21, 26].

### Flow cytometry analysis

For the stem cell phenotype identification, purified ERCs were obtained and stained with surface marker (CD29, CD45, CD90, and CD105) fluorescent antibody (ebioscience Inc., USA). In addition, in order to identify Gal-9 expression, ERCs and IFN-γ-pre-stimulated ERCs were harvested and incubated with Gal-9 monoclonal antibody (Abcam, USA) for 30 min. Following a washing step, Alexa Fluor®488-conjugated secondary antibody (Jackson ImmunoResearch Inc., USA) was added and incubated in the dark place for another 30 min, followed by flow cytometry analysis. All the procedures were carried out on ice, and no antibody was added into the control group.

In order to further evaluate the co-cultivation system of lymphocytes and ERCs in vitro, after collection and centrifugation, fluorescent antibody was adopted for staining directly. While for the spleens obtained in the in vivo experiments, it need to be gotten through the procedures with grinding, filtering with a 100-mesh filter, and lysing red blood cells, and then the single-cell suspension was obtained for the following staining. Fluorescent monoclonal antibodies (ebioscience Inc., USA) were used respectively for measuring the proportion of Th1 (CD4^+^IFN-γ^+^), Th17 (CD4^+^IL-17^+^), Treg (CD4^+^CD25^+^FoxP3^+^), and B cells (CD19^+^CD86^+^). In addition for detecting Th1 and Th17, cell stimulation cocktail (including phorbol-12-myristate-13-acetate (PMA), ionomycin, brefeldin A, and monensin) was added 5 h in advance to stimulate cytokine production and prevent its secretion, followed by fluorescent antibody staining. Furthermore, to evaluate Tim-3 change during the process, we also measured CD8^+^Tim-3^+^ and CD4^+^Tim3^+^ T cell proliferation*.*

In addition, for measuring the proportion of donor-specific antibody, 5 μl of the serum was collected from the recipient (B6, H-2^b^) at postoperative day (POD) 8. Serum dilution (1:20 dilution in PBS) was then co-cultivated with the splenocytes (5 × 10^5^ cells) obtained from the donor (BALB/c, H-2^d^) at 37 °C for 30 min, followed by staining with both anti-CD3-FITC and anti-IgG-PE (for IgG measurement), or anti-CD3-FITC and anti-IgM-PE antibody (for IgM measurement). In this method, donor-derived CD3 T cells were adopted to detect alloreactive antibody proportion, in case that the donor-specific antibody could bind through FC segment with Fc receptor owing cells (e.g., macrophages), to reduce the background interference [27].

### Enzyme-linked immunosorbent assay (ELISA)

As described above, P2-P5 generation ERCs and IFN-γ-stimulated ERCs were obtained separately, to measure Gal-9 expression. Furthermore, the supernatant, gathering from the in vitro ERC and splenocyte (after LPS stimulation) co-culture system, was also collected to detect the level of IgG and IgM. All the tests were carried out by using the produced ELISA kits (DAKEWE, Shenzhen, China) and following the procedures recommended by the manufacturer’s instructions.

### Heterotopic cardiac transplantation and experimental groups

Abdominal heterotopic cardiac transplantation was performed as previously described [28]. Briefly, the heart graft from BALB/c (H-2^d^) mice was slowly perfused with 1.0 ml of cold heparin (100 U) solution through the inferior vena cava. Then the superior vena cava, inferior vena cava, and pulmonary vein were separated and ligated, while the ascending aorta was retained and sutured on the recipient’s abdominal aorta (C57BL/6, H-2^b^) in an end-to-side anastomosis, and the pulmonary artery was anastomosed to the recipient’s inferior vena cava.

After resuscitation, B6 mice were randomly divided into 6 groups (*n* = 6): (1) untreated group: no special treatment was rendered after transplantation. (2) ERC treatment group: ERCs were administered intravenously (5 × 10^6^ cells) 24 h after cardiac transplantation. (3) Rapamycin (Rapa) treatment group: Rapa (Solarbio, Beijing, China) was dissolved in olive oil and injected subcutaneously at a dose of 2 mg/kg/day for consecutive 13 days after completion of the transplantation. (4) Gal-9-ERC treatment group: ERCs were pre-stimulated by IFN-γ for 72 h, and then digested, washed, and resuspended in PBS for injection (5 × 10^6^ cells, 24 h postoperation). (5) ERC and Rapa combination treatment group. (6) Gal-9-ERC and Rapa combination treatment group. The beating of the grafted heart was monitored daily by direct abdominal palpation. For the study of the survival, the observation end point was determined when the heart pulsation was completely ceased or at postoperative day 100 (POD100). For the evaluation of the treatment effect and immune status of different experimental groups, spleen, serum, and cardiac allografts were harvested respectively at POD8 and used for the following assessment.

### H&E staining

Cardiac allografts used for pathological analysis were obtained at POD8 and fixed in 10% formalin. Then the specimens were embedded in paraffin and sectioned into a 5-μm slice for hematoxylin-eosin (H&E) staining. Two pathologists double-blindly assessed the pathology changes of the graft, and the assessment criteria are as follows: with presence of vasculitis, thrombosis, hemorrhage, and lymphocyte infiltration. Pictures representing the average pathological changes in each group were selected for display.

### Real-time polymerase chain reaction (RT-PCR)

ERCs with or without IFN-γ stimulation were collected for Gal-9 measurement, and splenocyte suspensions from the co-culture system were obtained to determine the cytokine expression (IFN-γ, IL-17, and IL-35). Moreover, cardiac allografts were harvested at POD8 for detection of human-derived Gal-9 and mouse cytokines. Total RNA among the above cells or tissues were extracted respectively according to the manufacturer’s instructions (DP430, DP431, Tiangen Biotech Co. Ltd.). Then the purity and concentration of the extracted RNA were evaluated by a UV spectrophotometer at 260 and 280 nm. Following the evaluation, total RNA was immediately reverse transcribed into cDNA by using a FastKing one-step kit (KR106, Tiangen Biotech Co. Ltd). Then RT-PCR reaction was performed by SuperReal Color Premix kit (FP216, Tiangen Biotech Co. Ltd.) according to the manufacturer’s recommended procedure. The primer sequences used in this experiment are designed as follows:

**Table:** 

Gene	Primers (5′–3′)
GAPDH (human)	Forward: GGAGCGAGATCCCTCCAAAAT
	Reverse: GGCTGTTGTCATACTTCTCATGG
Galectin-9 (human)	Forward: GGACGGACTTCAGATCACTGT
	Reverse: CCATCTTCAAACCGAGGGTTG
GAPDH (mice)	Forward: AGGTCGGTGTGAACGGATTTG
	Reverse: TGTAGACCATGTAGTTGAGGTCA
IFN-γ (mice)	Forward: ATGAACGCTACACACTGCATC
	Reverse: CCATCCTTTTGCCAGTTCCTC
IL-17 (mice)	Forward: TTTAACTCCCTTGGCGCAAAA
	Reverse: CTTTCCCTCCGCATTGACAC
EBI3 (IL-35, mice)	Forward: CTTACAGGCTCGGTGTGGC
	Reverse: GTGACATTTAGCATGTAGGGCA
T-bet (mice)	Forward: AGCAAGGACGGCGAATGTT
	Reverse: GGGTGGACATATAAGCGGTTC
RORyt (mice)	Forward: GACCCACACCTCACAAATTGA
	Reverse: AGTAGGCCACATTACACTGCT
Foxp3 (mice)	Forward: CCCATCCCCAGGAGTCTTG
	Reverse: ACCATGACTAGGGGCACTGTA

Each specimen measurement was performed twice, and the housekeeping gene GADPH was used as a reference for normalization. The relative differences of target gene expression among different groups were analyzed by the comparative 2^−ΔΔCT^ method.

### Western blotting

ERCs with or without IFN-γ pre-stimulation were washed and lysed in the RIPA lysis buffer containing a protease inhibitor. The supernatant was then heated in a water bath, followed by measuring the concentration by a BCA kit. After quantifying the protein, the homogenization solution was then separated by SDS-PAGE and transferred to a PVDF membrane. Followingly, the obtained bands were blocked with BSA solution, and then incubated with primary antibody (Anti-human Gal-9, 1:2000; Anti-human Hsp90, 1:1000) and secondary antibodies (Anti-rabbit, 1:2000). At last, the bands were washed with TBST and developed with an ECL solution. The strips were photographed on the exposure platform, and fluorescence intensity was analyzed by ImageJ.

### Immunofluorescence staining

In order to analyze the expression of Gal-9 more intuitively, ERCs were crowded on the slides placed in a 6-well plate with or without IFN-γ stimulation (20 ng/ml, 72 h). Then these slides were taken out and fixed in 4% (w/v) paraformaldehyde, followed by permeabilizing in 0.1% Triton X-100. After blocking and washing, the slides were finally incubated with anti-human Gal-9 primary antibody (Abcam, USA) and Alexa Fluor® 488-conjugated secondary antibody respectively (Jackson ImmunoResearch Inc., USA). Lastly, these slides were mounted with DAPI containing antifade mountants (SouthernBiotech, USA) and photographed by fluorescence microscopy.

### Statistical analysis

All the data were presented as Mean ± SD, and cardiac survival analysis was conducted through the Kaplan-Meier cumulative survival method, and survival differences between groups were determined by the log-rank test. Differences among groups were assessed by using one-way analysis of variance (ANOVA) (groups ≧ 3) or unpaired *t* test (groups = 2). *P* value < 0.05 was considered statistically significant.

## Results

### Characterization of ERCs

**Fig. 1:**
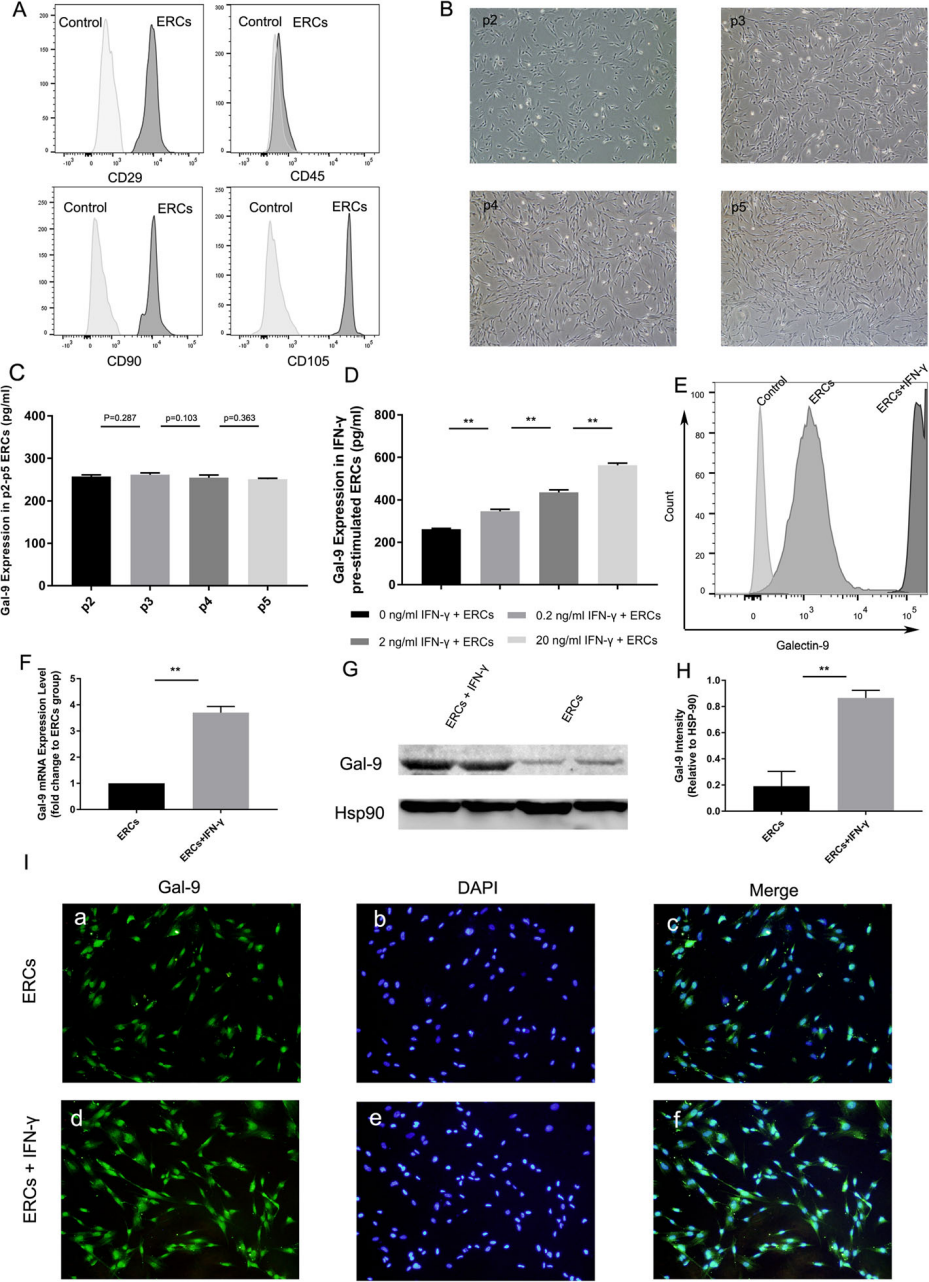
ERC characteristics and Gal-9 expression evaluation. **A** FACS analysis of ERC surface markers (CD29, CD45, CD90, and CD105). **B** Morphology of p2-p5 passage ERCs. **C** Gal-9 expression in p2-p5 ERCs were measured by ELISA, and there was no statistical difference among different generations (*n* = 3). **D** Gal-9 expression in ERCs was elevated by IFN-γ stimulation in a dose-dependent manner (*n* = 3). **E** Surface expression of Gal-9 in ERCs and IFN-γ-pre-stimulated ERCs (20 ng/ml, 72 h) were detected by flow cytometry. **F** Relative mRNA expression difference was analyzed between ERCs and IFN-γ-pre-stimulated ERCs (20 ng/ml, 72 h) by RT-PCR (*n* = 3). **G** Gal-9 protein expression differences in ERCs and IFN-γ-pre-stimulated ERCs (20 ng/ml, 72 h). **H** Gray value analysis based on western blot; Gal-9 intensity analysis was homogenized after comparing with HSP-90. **I** Immunofluorescence staining was performed to verify Gal-9 expression among ERCs and IFN-γ-pre-stimulated ERCs (20 ng/ml, 72 h). Gal-9 (green) and nuclei (blue) were merged together in (c) and (f). Differences among groups were assessed by using one-way analysis of variance (ANOVA) (groups ≧ 3) or unpaired *t* test (groups = 2).*p < 0.05, **p < 0.01. Abbreviations: ERC, endometrial regenerative cell; Gal-9, Galectin; p2-p5, passage 2-passage 5; wb, western blot; HSP-90, Heat shock protein 90

Purified ERCs could be easily characterized and identified by cell markers expressed on their surface. Based on the verified Abs for ERCs, this study demonstrated that ERCs were positive for CD29, CD90, and CD105, while negative for CD45 (Fig. 1A). Additionally, P2-P5 ERCs were collected and photographed. As shown in Fig. 1B, these cells exhibited a heterogeneous fibroblastic-like or spindle-shape morphology, and colony formation ability. In addition, for ERCs after passage 2, their average doubling time is 24 h, indicating that ERCs were also with a high proliferation rate.

### ERCs highly expressed Gal-9 after being stimulated by IFN-γ

In order to evaluate whether ERCs express Gal-9, cell lysates and culture supernatant of ERCs from passages 2–5 were harvested and analyzed by ELISA. The results demonstrated that ERCs indeed expressed Gal-9 (Fig. 1C and Supplementary Fig. 1), whereas the expression intensity was found with no statistical difference among different generations (Fig. 1C). Furthermore, after being stimulated by IFN-γ, it was found there was a significant increase of Gal-9 expression in ERCs and ERC culture supernatant, and this expression is increased by IFN-γ stimulation in a dose-dependent manner (Fig. 1D and Supplementary Fig. 1, *n* = 3, *p < 0.05, **p < 0.01).

To further verify Gal-9 expression difference between non-treated ERCs and IFN-γ-treated ERCs (IFN-γ stimulation, 20 ng/ml, 72 h), we performed flow cytometry analysis, which revealed that ERCs expressed Gal-9 on their cell surface, and with a higher expression of Gal-9 when compared to non-treated ERCs (Fig. 1E). RT-PCR and western blot analysis further demonstrated the above results at the gene and protein expression level (Fig. 1F, G). Statistical analysis and gray value measurements also confirmed that there was a higher expression of Gal-9 on ERCs after being stimulated by IFN-γ (Fig. 1F, H. *n* = 3, * *p* < 0.05, ** *p* < 0.01), while, to evaluate more intuitively, immunofluorescence staining was performed. When Gal-9 (green) and DAPI nuclei (blue) are merging together, it was easily found that IFN-γ-pre-stimulated ERCs expressed a higher amount of Gal-9 (Fig. 1I).

### Gal-9-ERCs suppressed T cell proliferation in vitro

**Fig. 2:**
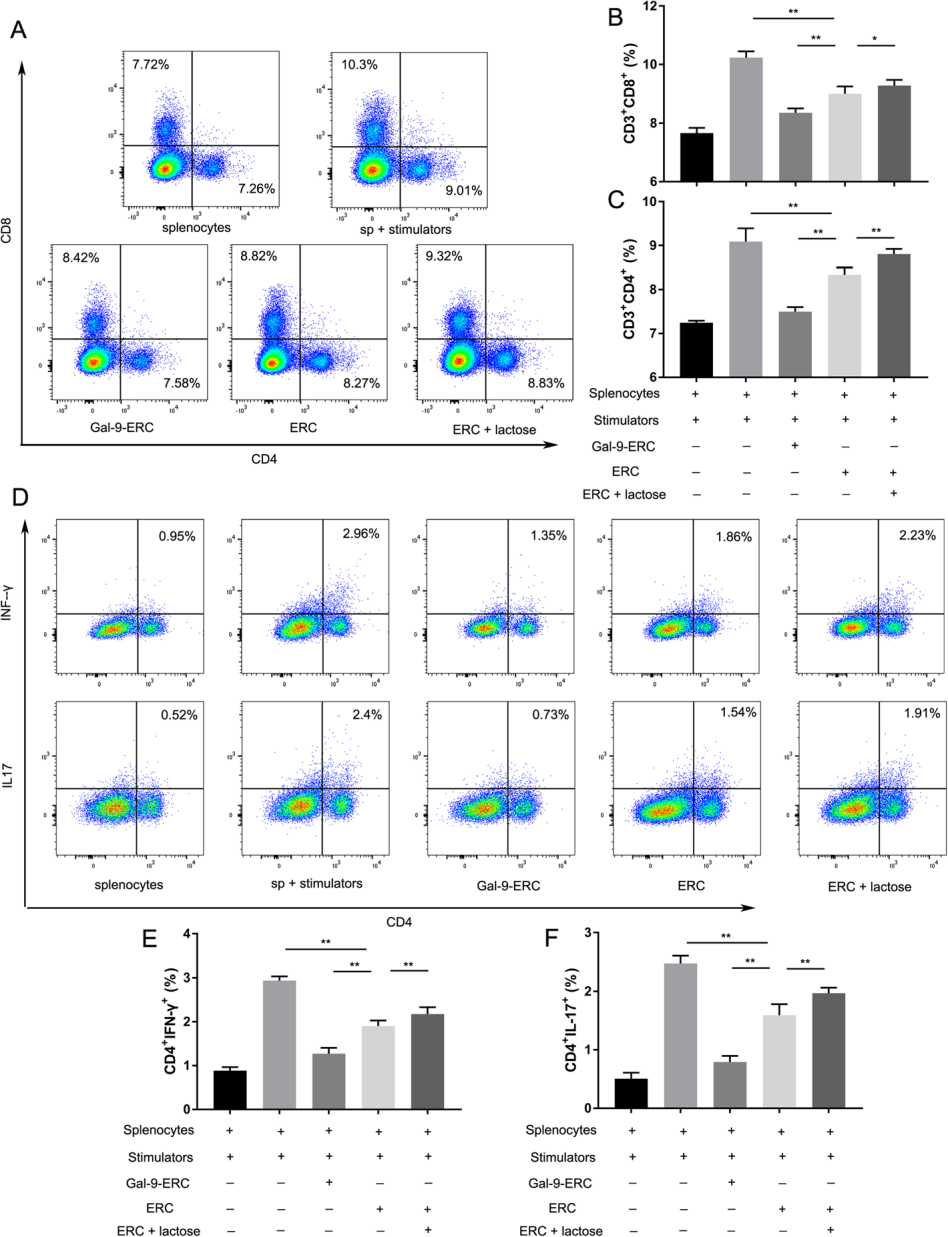
Gal-9-ERC suppressed T cell proliferation in vitro. Splenocytes (5 × 10^5^) were harvested from C57BL/6 mice and activated by the stimulants (IL-2 + anti-CD3/CD28 Abs). Then, ERC, Gal-9-ERC (IFN-γ pre-stimulate, 20 ng/ml, 72 h), and ERC + lactose were co-cultivated with the above activated splenocytes respectively. After 96 h of co-cultivation, the splenocytes were collected for FACS analysis. **a** Representative dot plots of CD4^+^T (CD3^+^CD4^+^) and CD8^+^T (CD3^+^CD8^+^) cells. **b**, **c** Percentage of CD8^+^T (CD3^+^CD8^+^) and CD4^+^T (CD3^+^CD4^+^) cells (*n* = 6). **d** Representative dot plots of Th1 (CD4^+^IFN-γ^+^) and Th17 (CD4^+^IL-17^+^) cells. **e**, **f** Percentage of Th1 (CD4^+^IFN-γ^+^) and Th17 (CD4^+^IL-17^+^) cells (*n* = 6). Differences among groups were assessed by using one-way analysis of variance (ANOVA).*p < 0.05, **p < 0.01. Abbreviations: ERC, endometrial regenerative cell; Gal-9-ERC, Galectin-9 high-expression ERC

To explore whether the modulatory effect of ERCs correlated with the Gal-9 expression, C57BL/6-derived splenocytes were collected and co-cultured with Gal-9-ERCs, ERCs, and ERCs+lactose (Gal-9 blocker) in vitro. The cell culture suspensions were subjected to flow cytometric analysis, and representative data for each group is shown in Fig. 2a. When the splenocytes were stimulated with IL-2 + anti-CD3/anti-CD28 Abs, the percentages of CD4^+^ T and CD8^+^ T cells increased significantly (splenocytes vs. splenocytes+stimulators, *p* < 0.01). However, co-culture with ERCs reduced the proliferative rate of stimulated CD4^+^ T and CD8^+^ T cells, with increased suppression in the Gal-9-ERC group (vs. ERC group, *p* < 0.01) (Fig. 2a–c). In contrast, in the lactose-ERC group, the regulatory effect of ERCs was significantly weakened (vs. ERC group, *p* < 0.01) (Fig. 2a–c). Of note, lactose is reported to competitively antagonize Gal-9 binding ligand, thus blocking Gal-9 immunomodulation effect [26].

In addition, to evaluate Tim-3^+^ changes during the process, we measured CD8^+^Tim-3^+^ and CD4^+^Tim3^+^ T cell proliferation in the co-culture system. We found that Tim-3 changes were accompanied with the proportion changes of CD8^+^ T and CD4^+^ T (Th1 and Th17) cells, indicating that Tim-3 is a stable ligand expressed on the above cells (Supplementary Fig. 2). Given the above results, it suggested that ERC participated in regulating activation and expansion of CD4^+^ T and CD8^+^ T cells, and the regulatory effect was enhanced by Gal-9 high expression and decreased after antagonism of Gal-9.

### Gal-9-ERCs reduced Th1/Th17 population and cytokine expression in vitro

**Fig. 3:**
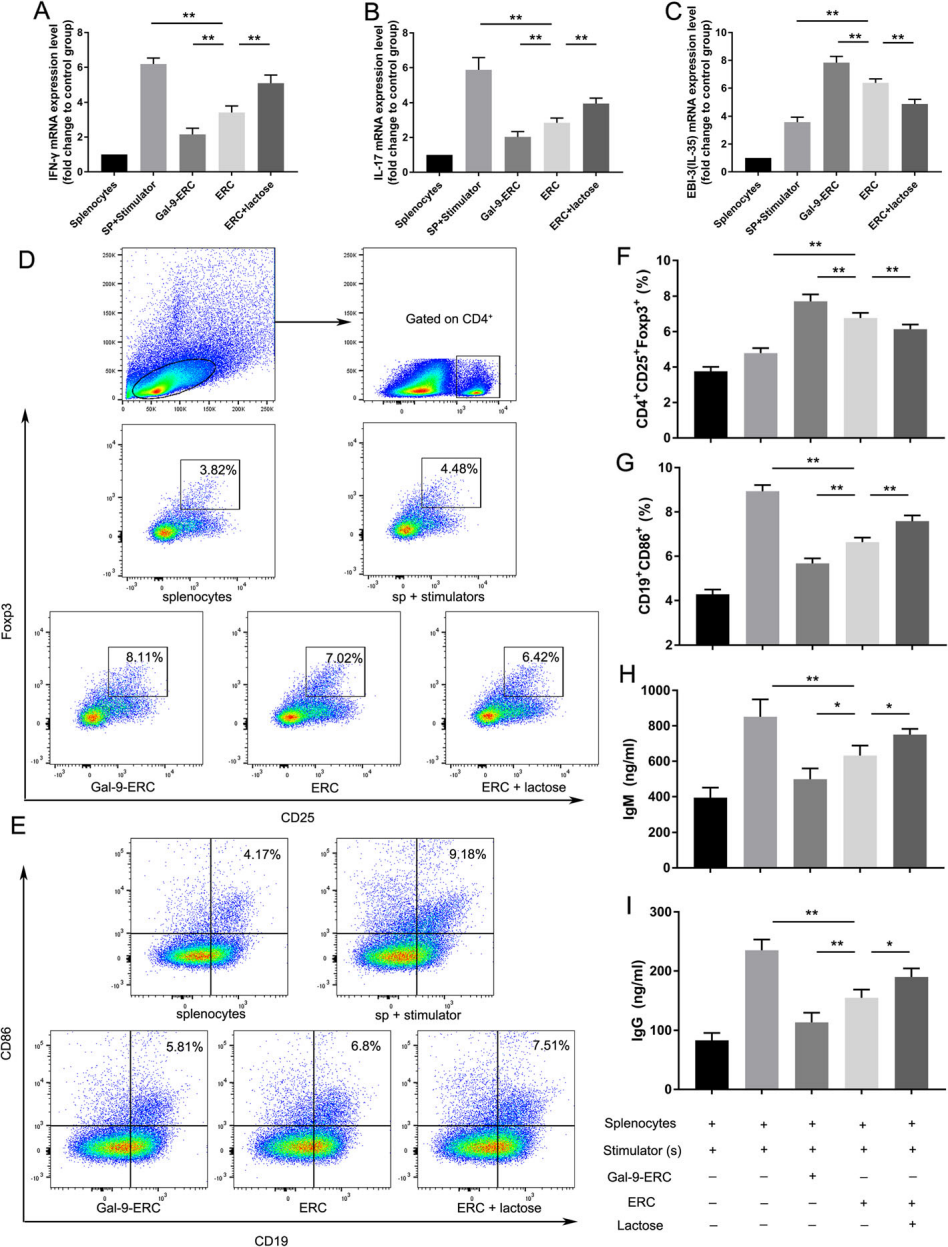
Gal-9-ERC promoted Tregs while inhibiting B cell activation in vitro. On account of the above co-culture experiments, total cell mRNA was extracted and used for RT-PCR analysis. IFN-γ (**a**), IL-17 (**b**), and EBI3 (IL-35) (**c**) mRNA relative expression differences were measured among different groups respectively (*n* = 6). **d** Representative dot plots of Treg (CD4^+^CD25^+^Foxp3^+^) cells, and the stimulant for Tregs was IL-2 + anti-CD3/CD28 Abs. Gating strategy was shown above, and all the following analysis were gated on CD4^+^ population. **e** Representative dot plots of activated B (CD19^+^CD86^+^) cells and stimulant for B cells is LPS. **f**, **g** Percentage of Treg (CD4^+^CD25^+^Foxp3^+^) and B (CD19^+^CD86^+^) cells (*n* = 6). **h**, **i** Furthermore, to evaluate antibody-producing plasma B cell levels, IgM (**h**) and IgG (**i**) in the culture supernatant were also collected and detected by ELISA (*n* = 6). Differences among groups were assessed by using one-way analysis of variance (ANOVA). *p < 0.05, **p < 0.01. Abbreviations: ERC, endometrial regenerative cell; Gal-9-ERC, Galectin-9 high-expression ERC; EBI3, Epstein-Barr Virus Induced 3; LPS, lipopolysaccharides

Gal-9 has been identified as the ligand for Tim-3, which is highly expressed on Th1 and Th17 cells. We next evaluated whether Gal-9-ERCs could assist in suppressing Tim-3^+^ T helper cells. As shown in Fig. 2d, both CD4^+^IFN-γ^+^ and CD4^+^IL-17^+^ cells increased significantly after adding stimulators (splenocytes vs. splenocytes+stimulators, *p* < 0.01). The percentage of CD4^+^IFN-γ^+^ and CD4^+^IL-17^+^ cells were decreased after co-culture with ERCs, and further reduced significantly in the Gal-9-ERC group (Fig. 2e, f). This decrease was reversed when lactose, the Gal-9 antagonist, was added to the mix, as determined by cytokine mRNA expression (Fig. 3a, b), which further indicates that Gal-9 is involved in regulating the number and function of Th1 and Th17 cells.

### Gal-9-ERCs enhanced Treg population and inhibited B cell activation

It has been previously reported that exogenous Gal-9 promoted iTreg differentiation and inhibited BCR signaling transduction [21]. Therefore, to further determine the effect of Gal-9-ERCs on Tregs and B cells in vitro, we stimulated splenocytes either with IL-2 + anti-CD3/anti-CD28 Abs for Treg induction, or with LPS for B cell activation, and then co-cultured these stimulated cells with ERCs. Results were analyzed by flow cytometry, and the gating strategy is shown in Fig. 3d. After gating the CD4^+^ cells, CD25^+^ and Foxp3^+^ double-positive cells (Tregs) were evaluated. As expected, the percentage of Tregs increased slightly after administrating IL-2 + anti-CD3/anti-CD28 Abs (splenocytes vs. splenocytes+stimulators, *p* < 0.05), while Treg population further increased in Gal-9-ERC group, but decreased in ERC + lactose group (Fig. 3f). mRNA expression of EBi-3, a component of IL-35, whose expression could indicate, at least in part, Treg activation, is significantly increased when co-cultured with Gal-9-ERCs (Fig. 3c).

We next evaluated CD19^+^CD86^+^ B cells and IgG/IgM production in the co-culture system. As shown in Fig. 3e, in the presence of LPS as stimulator, CD86 expression is increased in the splenocytes+stimulator group, reflecting an active state, which was downregulated in the ERC group. This alleviation was further strengthened in the Gal-9-ERC group, while abrogated in the ERC + lactose group (Fig. 3g, Gal-9-ERC group vs. ERC group, *p* < 0.01; ERC + lactose group vs. ERC group, *p* < 0.01). Moreover, on account that majority of LPS-treated B cells mainly differentiate into antibody-producing plasma B cells, we measured IgG/IgM antibody production in the supernatants of the co-culture system by ELISA. Consistently, both IgG and IgM production were significantly decreased in the Gal-9-ERC group (*p* < 0.01), while they were increased in the ERC + lactose group (*p* < 0.01), when compared with the ERC group. Given together, these data suggest that Gal-9-ERCs play a critical role in inhibiting B cell activation, downregulating costimulatory surface marker expression, and suppressing IgG/IgM antibody production.

### Gal-9-ERC-based therapy prolonged cardiac allograft survival

**Fig. 4:**
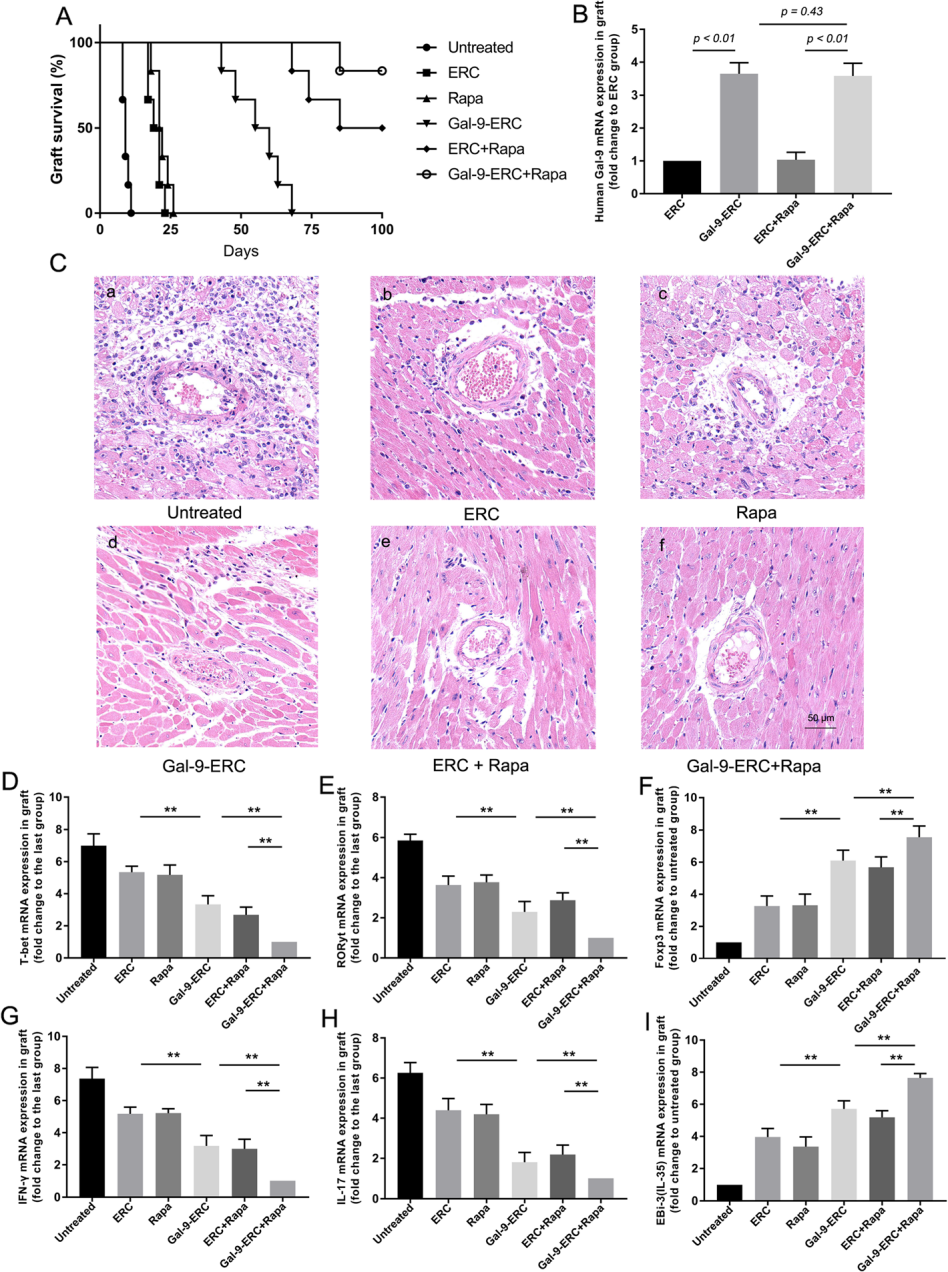
Gal-9-ERC-based therapy alleviated pathological manifestation and prolonged allograft survival time. **A** Heterotopic cardiac transplantations (BALB/c to B6, *n* = 6) were performed, and a different immunotherapy was administrated successively. The mean survival time (MST) among different groups was compared, and survival analysis was conducted through the Kaplan-Meier cumulative survival method. **B** After receiving treatments, human-derived Gal-9 mRNA expression were evaluated in the murine cardiac allografts (*n* = 6). **C** Histology of cardiac allografts from B6 recipients on POD8. Representative sections among each group was harvested and stained for H&E staining (× 200 magnification). **D–I** To evaluate CD4^+^ T cell deviations and pro-cytokine expressions in allografts, relative mRNA expression of T-bet (**D**), RORyt (**E**), Foxp3 (**F**), IFN-γ (**G**), IL-17 (**H**), and EBI-3 (**I**) were separately analyzed, indicating Th1, Th17, and Treg deviations in grafts (*n* = 6). Differences among groups were assessed by using one-way analysis of variance (ANOVA). *p < 0.05, **p < 0.01. Abbreviations: ERC, endometrial regenerative cell; Gal-9-ERC, Galectin-9 high-expression ERC; POD, postoperative day; Rapa, rapamycin

Base on striking inhibition of T and B cell activation by Gal-9-ERCs in vitro, to address if Gal-9-ERCs have the similar effect in vivo, we assessed the tolerogenic properties of Gal-9-ERCs in a BALB/c-to-C57BL/6 mouse cardiac transplantation model. Here, we found that ERC alone significantly prolonged mean survival time (MST) of cardiac allografts (MST in untreated group, 9.17 days ± 0.48; MST in ERC group, 19.67 days ± 0.99; *p* < 0.001, Fig. 4A). Treatment with Gal-9-ERCs resulted in a further increase of MST (56.16 days ± 3.84), when compared to the ERC monotherapy group (*p* < 0.001), whereas, although Gal-9-ERC treatment prolonged graft survival, it might not be ideal enough to transform as a promising candidate for clinical therapy, due to the absent longer-term effect on promoting allograft survival.

Therefore, we added rapamycin (Rapa), a wildly used immunosuppressant which was recognized by its effect on inhibiting dendritic cells (DCs) and T cell activation through binding to the mTOR target, to the Gal-9-ERC-treated group. As shown in Fig. 4A, supplementing Rapa (i.e., ERC+Rapa and Gal-9-ERC+Rapa) resulted in prolonging allograft survival in both groups. The MST in the Rapa monotherapy group was 22.0 ± 1.13 days, while the MST increased in both the ERCs+Rapa group (87.83 ± 5.37 days, *p* < 0.001) and the Gal-9-ERCs+Rapa group (97.5 ± 2.28 days, *p* < 0.001). Overall, these results suggest that Gal-9-ERC in combination of Rapa are more effective in prolonging the survival of cardiac allografts.

### Gal-9-ERC-based therapy improved human Gal-9 expression and alleviated pathological manifestation in cardiac allografts

To clarify whether Gal-9-ERC infusion promotes human ERC-derived Gal-9 expression in mouse cardiac grafts, RT-PCR was performed by using human Gal-9 primers. As shown in Fig. 4B, Gal-9 relative mRNA expression was increased in the Gal-9-ERC group (vs. ERC group, *p* < 0.01) and Rapa+Gal-9-ERC group (vs. Rapa+ERC group, *p* < 0.01). whereas there was no statistical difference between the Gal-9-ERC group and Rapa+Gal-9-ERC group, indicating that Gal-9-ERC, not Rapa, could promote Gal-9 expression in cardiac allografts.

In addition, to evaluate pathological alleviation mediated by Gal-9-ERC treatment, cardiac allografts were harvested on POD8 and evaluated by H&E staining (Fig. 4C). As shown, the untreated group show severe rejection, characterized by monocyte infiltration, interstitial hemorrhage, myocyte degeneration, and subtotal vascular luminal obliteration, whereas pathological signs of rejection are decreased in the ERC (Fig. 4C (b)) and Rapa (Fig. 4C (c)) monotherapy groups, manifested by milder monocyte infiltration in the ERC-treated group and moderate myocyte degeneration in the Rapa-treated group. These pathological changes were further decreased in the Gal-9-ERC-treated group (Fig. 4C (d)) and ERC + Rapa-treated group (Fig. 4C (e)). And eventually, in Gal-9-ERC in combination with the Rapa group (Fig. 4C (f)), it shows almost normal tissue structure, with scarce signs of rejection. Overall, these manifestations suggested that Gal-9-ERC-based therapy was prerequisite in preventing grafts from rejection.

### Gal-9-ERC-based therapy modulated CD4^+^ cell differentiation in cardiac allografts

To evaluate whether Gal-9-ERC-based therapy has a modulatory effect on CD4^+^ cells in cardiac allografts, as observed in vitro, we measured mRNA expression of T-bet, RORyt, and Foxp3, which were recognized as specific transcription factors for Th1, Th17, and Treg cells respectively. As shown in Fig. 4D–F, T-bet and RORyt mRNA expressions were the highest in the untreated group and decreased in the Gal-9-ERC group, when compared with the ERC group (*p* < 0.01). Moreover, these expressions further decreased in the Gal-9-ERC + Rapa group (vs. Gal-9-ERC group, *p* < 0.01; vs. Rapa+ERC group, *p* < 0.01). Conversely, Foxp3 mRNA expression increased in the Gal-9-ERC group (vs. ERC group, *p* < 0.01) and was the highest in the Gal-9-ERC + Rapa group (vs. Gal-9-ERC group, *p* < 0.01; vs. Rapa+ERC group, *p* < 0.01).

We next evaluated cytokine expression in allografts, including IFN-γ, IL-17, and EBI-3 (IL-35), which are secreted by Th1, Th17, and Treg cells respectively. Consistently, changes in expression of these cytokines reflected what we observed in the changes of expression levels of the transcriptional factors (Fig. 4G–I). Taken together, these results reveal that combination therapy of Gal-9-ERC and Rapa could modulate allograft CD4^+^ cell differentiation, which resulted in reducing Th1 and Th17 cells and increasing the proportion of Treg cells.

### Gal-9-ERC-based therapy inhibited CD4^+^ T and CD8^+^ T cell response while promoting Treg generation

**Fig. 5:**
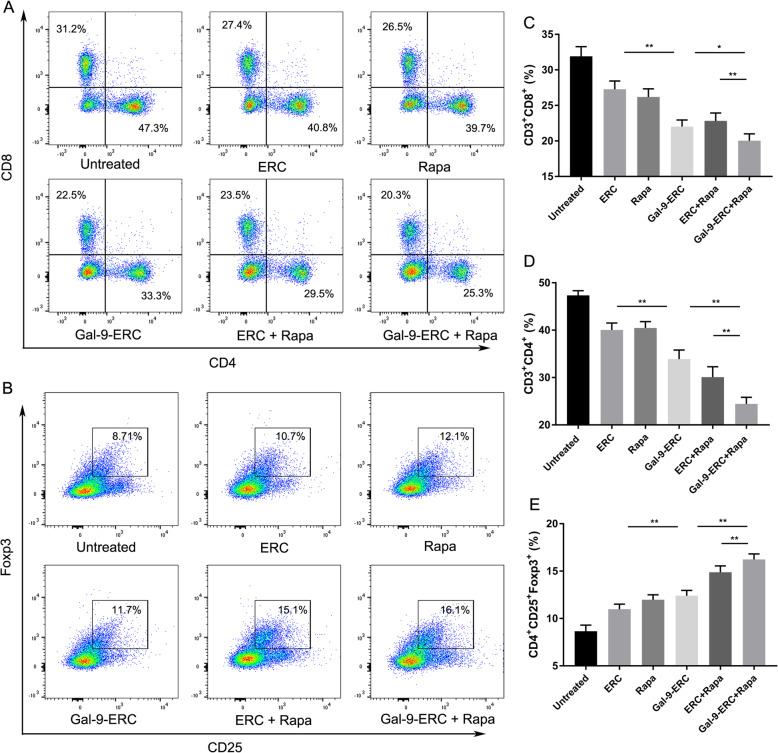
Gal-9-ERC-based therapy inhibited CD4^+^ T and CD8^+^ T cell response while promoting Treg generation. Immune cells (CD4^+^ T and CD8^+^ T) activation and proliferation reflect the severity of acute cellular rejection (ACR). Splenocytes from B6 recipients among each group were harvested on POD8 and then stained for flow cytometry analysis. **a** Representative dot plots of CD4^+^ T (CD3^+^CD4^+^) and CD8^+^ T (CD3^+^CD8^+^) cells. **b** Representative dot plots of Treg (CD4^+^CD25^+^Foxp3^+^) cells. **c–e** Percentage of CD8^+^ T (CD3^+^CD8^+^), CD4^+^ T (CD3^+^CD4^+^) and Treg (CD4^+^CD25^+^Foxp3^+^) cells (*n* = 6). Differences among groups were assessed by using one-way analysis of variance (ANOVA). *p < 0.05, **p < 0.01. Abbreviations: ERC, endometrial regenerative cell; Gal-9-ERC, Galectin-9 high-expression ERC; POD, postoperative day; Rapa, rapamycin

CD4^+^ T and CD8^+^ T cell proliferation and infiltration reflect the severity of acute cellular rejection (ACR), which is an important manifestation at the early stage of rejection. Moreover, Tregs hamper the proliferation of CD4^+^ T cells, CD8^+^ T cells, and DCs, thus promoting transplant tolerance. To evaluate the effect of Gal-9-ERC-based therapy in modulating effector T cell and Treg function, we harvested the splenocytes on POD8 and performed flow cytometric analysis. As shown in Fig. 5a, representative figure in each group was selected and gated on CD3^+^ cells. The percentage analysis is shown in Fig. 5c, d (CD3^+^CD8^+^ cells, Fig. 5c; CD3^+^CD4^+^ cells, Fig. 5d). Briefly, in the untreated group, percentages of CD4^+^ T and CD8^+^ T cells were the highest, and it tended to reduce in the Gal-9-ERC group (vs. ERC group, *p* < 0.01). Moreover, these percentages were further reduced in the Gal-9-ERC + Rapa group, when compared with the Gal-9-ERC (*p* < 0.01) and ERC + Rapa groups (*p* < 0.01) respectively.

In addition, we also measured CD4^+^CD25^+^Foxp3^+^ Tregs in splenocytes (Fig. 5b), and the percentage analysis was shown in Fig. 5e. As expected, the proportion of Tregs was increased when infused with Gal-9-ERC (vs. ERC group, *p* < 0.01). And it was further increased in the Gal-9-ERC + Rapa group (vs. Gal-9-ERC group, *p* < 0.01; vs. ERC + Rapa group, *p* < 0.01). Collectively, these data indicated that Gal-9-ERC-based therapy could suppress CD4^+^ T and CD8^+^ T cell proliferation, while promoting Tregs in the recipients.

### Gal-9-ERC-based therapy modulated T helper cell percentages

**Fig. 6:**
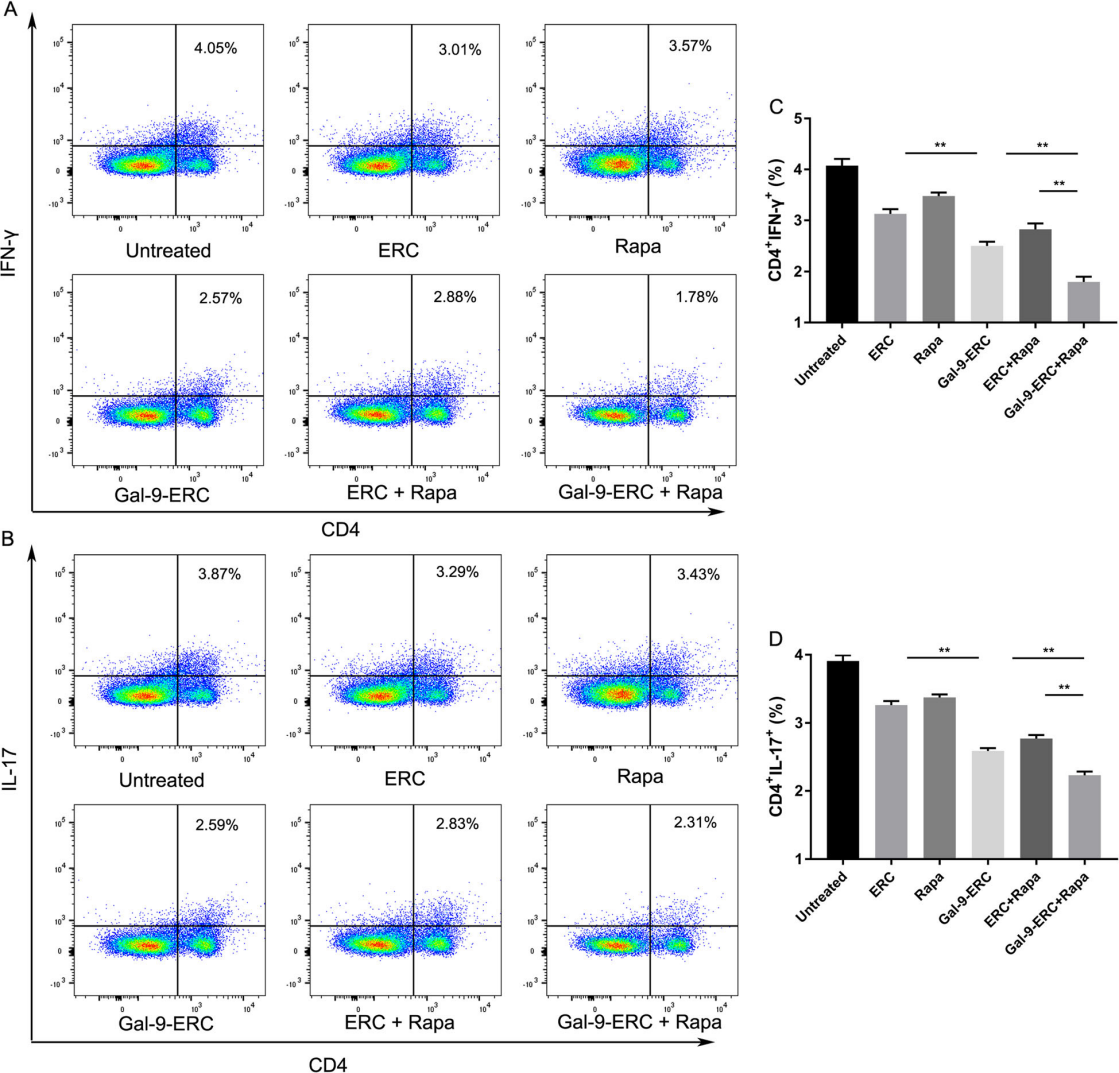
Gal-9-ERC-based therapy modulated T helper cell percentages. As observed in vitro experiment, and transcription factor expression differences in allografts, we would like to further evaluate Gal-9-ERC-based therapy in modulating T helper cells. Splenocytes were harvested on POD8 and stained for FACS. **a**, **b** Representative dot plots of Th1 (CD4^+^IFN-γ^+^) and Th17 (CD4^+^IL-17^+^) cells. **c**, **d** Percentage of Th1 (CD4^+^IFN-γ^+^) and Th17 (CD4^+^IL-17^+^) cells (*n* = 6). Differences among groups were assessed by using one-way analysis of variance (ANOVA). *p < 0.05, **p < 0.01. Abbreviations: ERC, endometrial regenerative cell; Gal-9-ERC, Galectin-9 high-expression ERC; Rapa, rapamycin

We next assessed whether Gal-9-ERC-based therapy could also modulate T helper cell populations, similarly to what we observed with the mRNA expression levels of transcription factor and cytokine expression changes in allografts. We stained the splenocytes and measured the percentages of Th1 (CD4^+^IFN-γ^+^) and Th17 (CD4^+^IL-17^+^) cells (Fig. 6a, b). The differences of cell percentages among each group were analyzed and then displayed on Fig. 6c, d. Collectively, both CD4^+^IL-17^+^ and CD4^+^IFN-γ^+^ cell percentages decreased in the Gal-9-ERC group (vs. ERC group, Th1, *p* < 0.01; Th17, *p* < 0.01), while these two populations were further reduced in the Gal-9-ERC + Rapa group (vs. Gal-9-ERC group, Th1, *p* < 0.01; Th17, *p* < 0.01; vs. ERC + Rapa group, Th1, *p* < 0.01; Th17, *p* < 0.01). Taken together, these data, in addition with the in vitro co-culture results and transcription factor changes in allografts, suggest that Gal-9-ERC-based therapy could decrease the number of Th1 and Th17 cells and thus assist in prompting allograft survival.

### Gal-9-ERC-based therapy inhibited B cell activation and donor-specific antibody (DSA) production

**Fig. 7:**
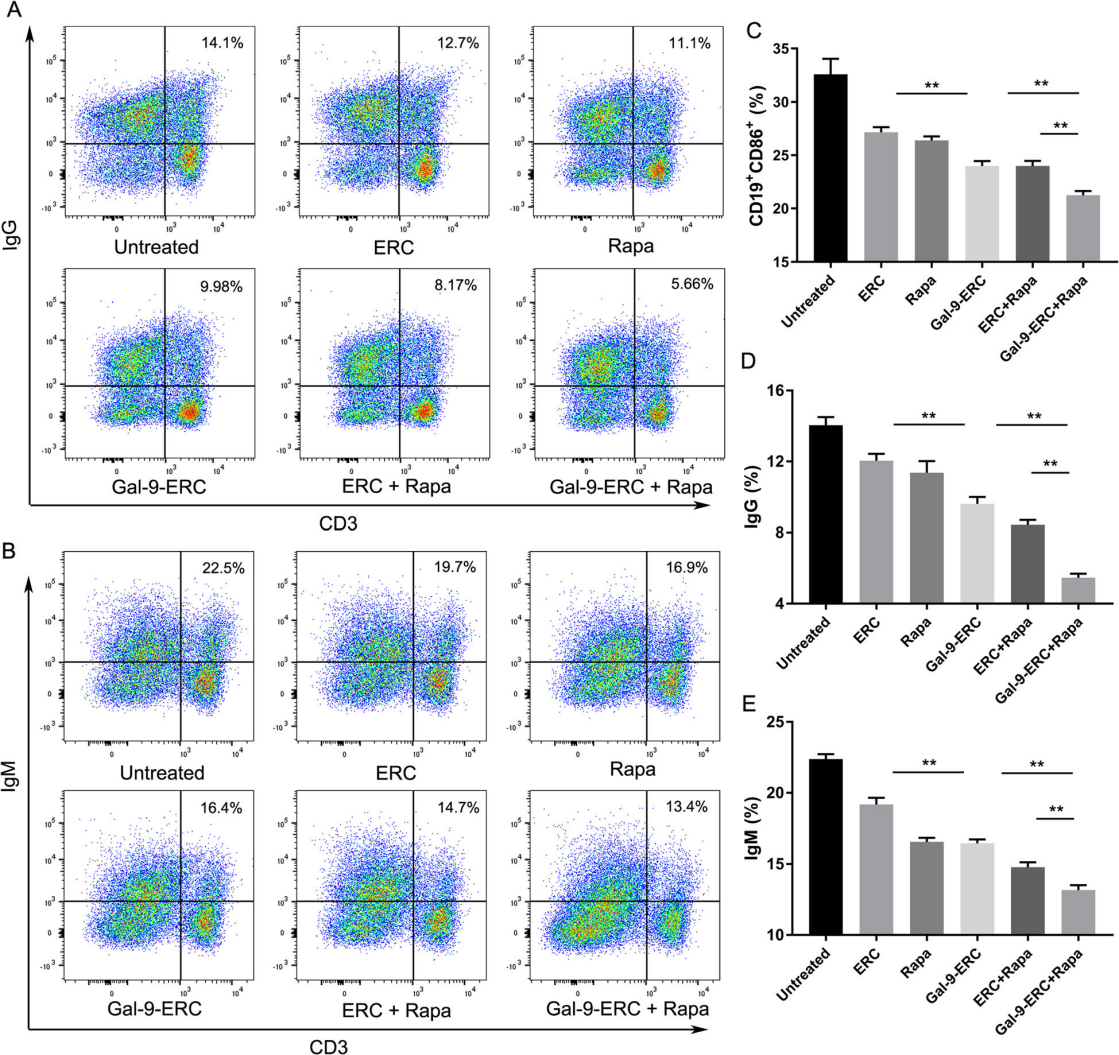
Gal-9-ERC-based therapy inhibited B cell activation and donor-specific antibody production. Once B cells activated, they can change into antigen-presenting type cells with co-stimulator expression or might transform into antibody-producing plasma cells, which play a vital role in mediating antibody-mediated rejection (AMR). Here, we measured donor-specific IgG and IgM, using the donor-derived splenocytes co-culturing with recipient serum (1:20 dilution) at 37 °C for 30 min. Then, donor CD3^+^T cell reactive IgG and IgM were measured by FACS analysis respectively. **a**, **b** Dot plots of CD3^+^IgG^+^ and CD3^+^IgM^+^. **c** Differential inhibition of B cell maturation/costimulatory surface marker expression. **d**, **e** Percentage of CD3^+^IgG^+^ and CD3^+^IgM^+^ in each group (*n* = 6). Differences among groups were assessed by using one-way analysis of variance (ANOVA). *p < 0.05, **p < 0.01. Abbreviations: ERC, endometrial regenerative cell; Gal-9-ERC, Galectin-9 high-expression ERC; Rapa, rapamycin

Once B cells are activated, they can change to either antigen-presenting cells, such as CD19^+^CD86^+^ B cells, or they might transform into antibody-producing plasma B cells, which play a vital role in antibody-mediated rejection (AMR). Here, we measure CD19^+^CD86^+^ B cells in the recipient spleens on POD8 by FACS (*data not shown*), and the differences are shown in Fig. 7c. The percentage of CD19^+^CD86^+^ B cells decreased significantly in the Gal-9-ERC group (vs. ERC group, *p* < 0.01), and it was further decreased in the Gal-9-ERC + Rapa group (vs. Gal-9-ERC group, *p* < 0.01; vs. ERC + Rapa group, *p* < 0.01), indicating that Gal-9-ERC-based therapy has a modulated effect on inhibiting B cell activation.

**Fig. 8:**
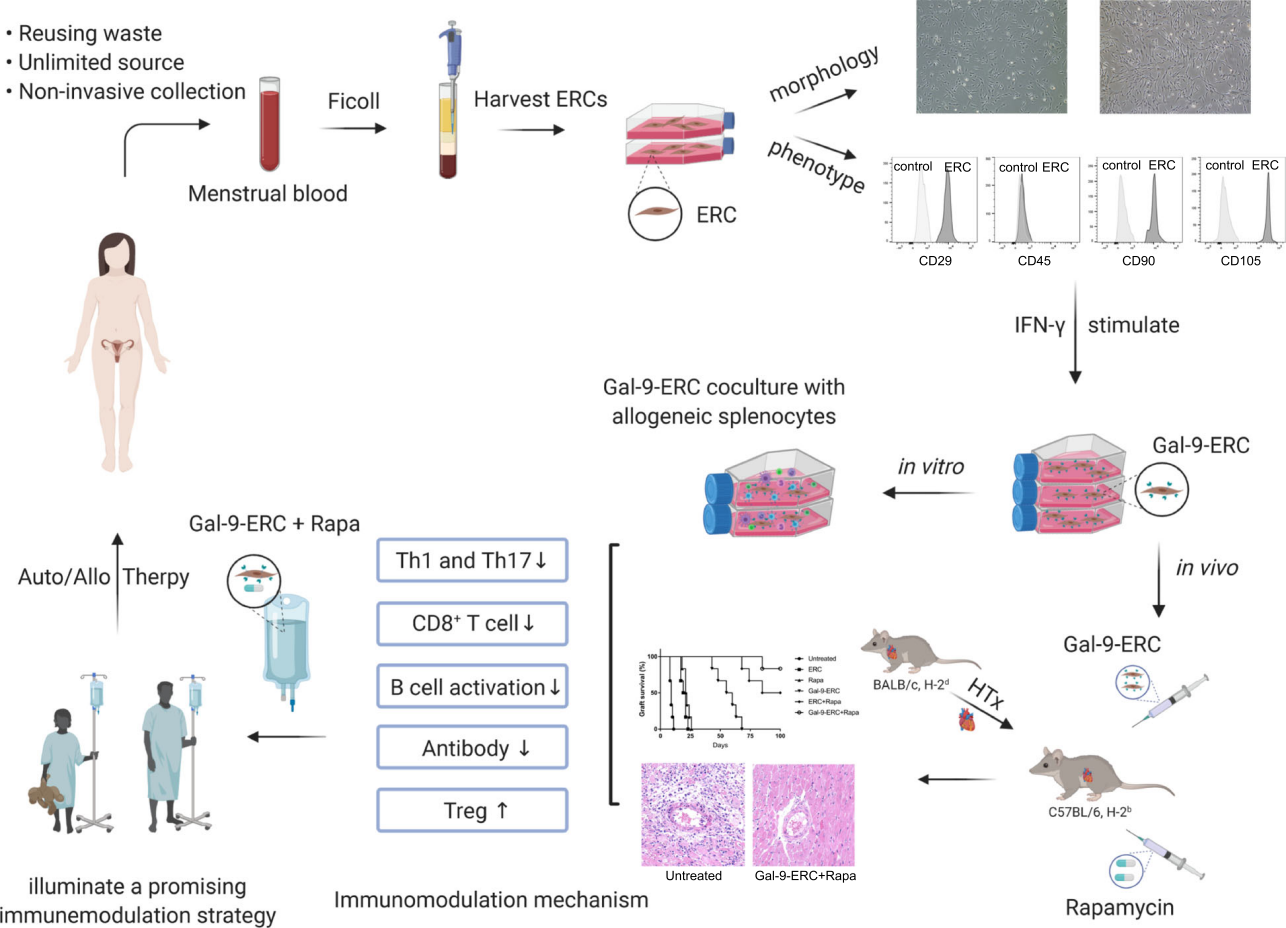
Isolation, cultivation, and potential clinical application of ERCs. Endometrial regenerative cells (ERCs), which are mesenchymal-like stem cells, were collected from a volunteer’s menstrual blood and identified as a new candidate for immune regulation. It has the advantages of reusing human waste, unlimited source, non-invasive collection method, and easy to large-scale expansion. In this study, we showed for the first time that ERCs could express Gal-9 and found that Gal-9-ERC-mediated therapy could assist in suppressing allogeneic Th1 and Th17 cell response, inhibiting CD8^+^ T cell proliferation, abrogating B cell activation, decreasing donor-specific antibody production, and promoting Tregs both in vitro and in vivo. These findings revealed that Gal-9 was required for ERCs to induce long-term cardiac allograft survival, which provides a novel idea for supplementing the ERC immunoregulatory mechanism and also offers a promising immunomodulation strategy to be verified in the clinical settings (created using www.biorender.com software)

In addition, we also measured donor-specific IgG and IgM antibody levels, as previously described [27]. Briefly, donor-derived splenocytes (BALB/c, H-2^d^) were collected to co-culture with the serum obtained from recipients (C57BL/6, H-2^b^, 1:20 dilution) at 37 °C for 30 min. Then, CD3^+^IgG^+^ or CD3^+^IgM^+^ were analyzed respectively to verify alloreactive antibody proportions. Representative figures are shown in Fig. 7a, b. Accordingly, the percentage analysis is shown in Fig. 7d, e. It was obvious that donor-specific IgG^+^ and IgM^+^ were both reduced in the Gal-9-ERC treatment group (vs. ERC group, IgG^+^, *p* < 0.01; IgM^+^, *p* < 0.01), indicating that Gal-9-ERC treatment interrupted with donor-specific antibody formation, whereas this alleviation effect was further strengthened in the Gal-9-ERC + Rapa group, when compared with the Gal-9-ERC group (IgG^+^, *p* < 0.01; IgM^+^, *p* < 0.01) and ERC + Rapa group (IgG^+^, *p* < 0.01; IgM^+^, *p* < 0.01). Given the above results, it suggests that Gal-9-ERC-based therapy could inhibit B cell activation and block donor-specific antibody production.

## Discussion

Uncontrolled immune rejection following transplantation, in addition with the numerous side effects caused by immunosuppressants is still a limiting factor in the long-term survival of the transplanted grafts [4]. ERCs, derived from the menstrual blood, are recognized as a new type of stromal stem cell. They get rid of the deficiencies in traditional invasive acquisition methods, and own the advantages in modulating immune response. In addition, ERCs have also been shown with the following benefits, such as abundant resource, non-invasive collection methods, and easy large-scale expansion capability [11, 29]. We have previously documented that ERCs could be affected by the chemokine SDF-1/CXCR4 and could accumulate at the injury site, thus mediating the long-term survival of the grafts [13]. However, the underlying immunomodulation mechanism of ERCs still needs to be elucidated.

It is widely recognized that ERCs could modulate the immune response through interacting with various immune cells, such as T cells, B cells, DCs, NK cells, and Tregs [12]. ERCs were also demonstrated to suppress immune reaction by paracrine mediators, including IL-10, IL-27, PGE-2, PDL-1, IDO, and COX-2. However, these findings are still not comprehensive and could not explain the reason for the enhancement of ERC immunoregulation ability after being stimulated by IFN-γ [30]. Based on our preliminary data, we found that ERCs could express Gal-9, and its expression could be enhanced by IFN-γ stimulation in a dose-dependent manner.

Gal-9, a member of galectin family, is the ligand for T cell immunoglobulin mucin-3 (Tim-3), which is expressed on the surface of CD8^+^ T, T helper type 1 (Th1), and a portion of Th17 cells [15]. Through Gal-9-Tim-3 interaction, Gal-9 could negatively regulate Th1 immunity, suppress the generation of Th17 cells, and inhibit CD8^+^ T cell response [18, 19]. Additionally, recombinant Gal-9 was also documented in abolishing BCR signaling transduction and in regulation of B cell activation [20, 21]. Further experiments also suggested that exogenous Galectin-9 was required for iTreg differentiation and maintenance [22]. Giving its extraordinary immunomodulation effect, Gal-9 was also used in prolonging the survival time of skin grafts and vascularized organs [31, 32]. Interestingly, it was found that recombinant Gal-9 indeed played a role in modulating the recipient’s immune system by suppressing Th1 cell and Th17 cell responses [33].

However, we hold that short-term recombinant Gal-9 administration might not be as effective as ERC-based cell therapy, which provides an alternative source for synthesizing and secreting Gal-9 continuously. Besides, ERCs were also verified with the ability to accumulate at inflamed sites via chemotaxis, which would enable ERC-based cell therapy to be much more specific and reliable [11, 13]. Additionally, previous studies also showed that ERCs possess a remarkable ability to differentiate into various cell lines, which enable its possible involvement in promoting injury repair [12, 34]. Given above, it suggests that ERC-based cell therapy is promising, and the Gal-9 mediated immunomodulation effect is worth exploring.

Accordingly, from multiple perspectives, we verified that ERCs expressed Gal-9 and found this expression could be enhanced by IFN-γ stimulation persistently and stably. Moreover, it is further confirmed by the flow cytometry and immunofluorescence results, which indicates that ERCs highly expressed Gal-9 after stimulating with IFN-γ, both on the cell membrane and cytoplasm.

To evaluate Gal-9-ERC-based therapy on modulating the immune response, we infused Gal-9-ERC into the recipients who received a cardiac allograft on POD1. As expected, Gal-9-ERC infusion prolonged the cardiac survival time when compared with ERC monotherapy. However, although this treatment significantly prolonged allograft survival, it is still without long-lasting effects and might not be ideal enough to transform as a promising candidate for clinical therapy. Here, we knew that Gal-9, besides having an effect on suppressing T and B cell activity, could also deliver proinflammatory signals to dendritic cells (DCs), which in turn would promote the formation of mature antigen-presenting cells (APCs) and accelerate rejection. Therefore, we proposed that Rapamycin (Rapa), a wildly used immunosuppressant, could be used to supplement Gal-9-ERC-based therapy. Rapamycin was wildly recognized for its ability in inhibiting DCs and T cell activity through binding to the mTOR target [35, 36], and thus might be with an effect on abrogating Gal-9-induced DC activation and promoting a long-term graft survival. In this study, we show that, in fact, Gal-9-ERC in combination with Rapa administration dramatically improved the graft survival time, which is better than ERC alone, ERC + Rapa, and Gal-9-ERC therapy.

Concerning the outstanding effect of Gal-9-ERC-based therapy, we further evaluated its immune modulation role in Th1 and Th17 cell deviations, according to in vitro and in vivo experiments. Th1-mediated immune response has been widely considered participating in acute allograft rejection. IFN-γ, which is secreted by Th1 cells, has been demonstrated to be elevated in the serum and in the graft, correlating with graft rejection in a number of clinical studies [37, 38]. Here, in our experiment, we found that Gal-9-ERC could suppress Th1 cell activation and IFN-γ expression in vitro. Moreover, when Gal-9-ERC, in combination with Rapa, were given to recipients, it was found with a decreased Th1 proportion in recipient splenocytes, paralleling with a downregulation of T-bet and IFN-γ mRNA expression in allografts. Giving the dominant role of Th1 in promoting rejection, this suppressing effect by Gal-9-ERC was thought to possibly contribute to prolonging the graft survival time, at least in part, by inactivating Th1 response and inhibiting IFN-γ expression.

Similar evidence exists for Th17 cells. IL-17, a cytokine produced by Th17 cells, has been implicated in the process of rejection and detected in human renal biopsies which was undergoing acute rejection, but not seen in normal kidney biopsies [39]. In addition, blocking IL-17 in a rat cardiac transplantation model has been found with a significant increase in graft survival time [40]. IL-17 mRNA has also been found elevated in bronchoalveolar lavage fluid from acute lung rejection patients, when compared with those without rejection [41]. Previously, Gal-9 has been found with an effect on suppressing the generation of Th17 cells through Gal-9-Tim-3 interaction.

Accordingly, in our study, we hypothesized that Gal-9-ERC would possess a similar effect on Th17 cells, attributed to the Gal-9 ligand. From our in vitro co-culture experiments, we found that Gal-9-ERC could suppress Th17 activation and inhibit IL-17 mRNA expression. Concurrently, from our in vivo data, we found Gal-9-ERC infusion to the recipient could inhibit Th17 cell response among splenocytes. We also found a decrease in IL-17 and RORyt mRNA expression in cardiac allografts, where RORyt is known as Th17-specific transcription factor. Given above, it was found Gal-9-ERC-based therapy could suppress Th17 cell activation and IL-17 expression, which might be realized by Gal-9 ligand. Besides, the Gal-9-ERC + Rapa combination group was found further alleviating Th17 activation, indicating that Rapa might have a synergistic role with Gal-9-ERC, rather than antagonistic effect, in inhibiting Th17 response.

CD4^+^ T and CD8^+^ T cells are believed actively involving in ACR. Giving the process of allo-recognition, donor-derived major histocompatibility complex (MHC) antigens would be presented to recipient T lymphocytes. Once recipient T cells are active, CD4^+^ Th1 and CD4^+^ Th17 cells could facilitate CD8^+^ T cell differentiation or directly promote graft rejection, by cell-to-cell contact or by producing effector cytokines, such as IFN-γ and IL-17 [42]. Then, undergoing clonal expansion, CD8^+^ T cells could differentiate into cytotoxic T lymphocytes, which would migrate to allograft and initiate tissue destruction [43]. Given above, in our study, we also measured CD4^+^ T and CD8^+^ T cell proportions when co-cultured with Gal-9-ERC in vitro. Moreover, the percentage of total CD4^+^ T and CD8^+^ T cells in recipient splenocytes were also evaluated. Consistently, from both in vitro and in vivo experiments, Gal-9-ERC-based therapy was found to inhibit CD4^+^ T and CD8^+^ T cell response. Nevertheless, CD8^+^ T cells might be inhibited through two distinct pathways, including the decreased IFN-γ secretion or Gal-9-Tim-3-mediated direct suppression.

Regulatory T cells (Tregs) have been shown to play a major role in the induction and maintenance of tolerance [44]. Multiple studies, focusing on the deletion or adoptive transfer of Tregs, have verified the critical role they play in modulating the immune response [45]. Here, we also evaluated Gal-ERC effect on Tregs. Through the in vitro co-culture experiments, we found that Gal-ERC could promote Treg proliferation and IL-35 mRNA expression. We further show that recipients treated with Gal-9-ERC and Rapa exhibit an increased proportion of Treg in splenocytes and IL-35 expression in allografts. Coincidentally, these findings were in line with other previous reports, which indicated that ERCs and Rapa could independently promote Tregs [13, 46].

In addition to looking at T effector cell activity, we also analyzed the effect of Gal-9-ERC therapy on antibody-mediated rejection (AMR), widely believed to contribute to chronic allograft vasculopathy (CAV), and thus limiting long-term survival of the transplanted grafts [27]. B cells were thought to play a critical role in mediating AMR. Once B cells interact with donor-derived HLA antigens, they can differentiate into antigen-presenting B cells (CD19^+^CD86^+^), which could trigger critical costimulatory signals to T cells when engaged with CD28 on T cell surface, or they may transform into plasma cells that produce donor-specific antibodies (DSA), thus facilitating AMR. Regrettably, current strategies for the treatment of AMR are limited [47].

Gal-9 was discovered by its effect on abolishing BCR signal transduction, and ERCs were also reported with a role in alleviating AMR. Thus, in this study, to evaluate Gal-9-ERC effect on AMR, we measured antibody expression changes in co-culture experiments and recipient serum. Collectively, we found there was a decrease in IgM and IgG antibody expression. In addition, we also found that the number of CD19^+^CD86^+^ B cells decreased in the co-culture system and in treated recipients. Taken together, this indicates that B cell activation may be one of the targets inhibited by Gal-9-ERC-mediated therapy.

On account of the outstanding immunomodulation role of Gal-9-ERC, to evaluate whether this effect was mainly mediated by Gal-9, we further measured human Gal-9 mRNA expression in murine allografts. As expected, we found there was still a higher Gal-9 mRNA expression in the treatment group on POD8, which was involved with Gal-9-ERC, indicating that Gal-9 retained higher expression. It was still not clear whether this higher expression was induced by the existing ERCs. However, it might help explain the reason for immune suppression and pathological manifestation changes in the allografts. In addition, in our study, the human-derived Gal-9 was found to function well in a mouse model, which was consistent with other reports [32], suggesting that Gal-9 is a highly conserved protein.

Given together, the results obtained from the current study are encouraging and promising. However, the detailed pathways associated with Gal-9-Tim-3 and Gal-9-IgM-BCR in this transplantation model require further elucidation. In the previous studies, *Zhuo* et al. have reported that Gal-9-TIM-3 interactions could activate downstream NF-κB and AKT pathways, inducing Th cell apoptosis [48, 49]. In addition, it has also been reported that the increased expression of Gal-9 was associated with STAT and JNK pathways [50]. *Anh* et al. found that Gal-9 could merge pre-existing nanoclusters of IgM-BCR, immobilize IgM-BCR, and relocalize IgM-BCR together with the inhibitory molecules CD45 and CD22, thus regulating B cell signaling [20, 21]. Thus, whether Gal-9, secreted by ERCs, would have the similar mechanism in the cardiac transplantation model still needs further evaluation.

In our present study, we focus on antagonizing or enhancing Gal-9 expression in ERCs by a lactose antagonist or IFN-γ pre-stimulation, respectively. We have analyzed that inhibitory or immunoregulatory effect of ERCs, which is, at least in part, mediated by Gal-9. Furthermore, the in-depth studies in the evaluation of therapeutic effects of Gal-9-gene-modified ERCs on cardiac allograft model are warranted.

In this study, we have shown for the first time that ERCs could express Gal-9 and found that Gal-9-ERC played a major role in immune modulation, which would provide a novel idea for supplementing the ERC immunoregulatory mechanism and also lay a foundation for the later experiment verification (Fig. 8). Furthermore, when we administered Gal-9-ERC to the recipients, we discovered a persisting enhanced Gal-9 mRNA expression in allografts, indicating that Gal-9-ERC treatment could promote Gal-9 expression persistently, which might surpass single-dose recombinant Gal-9 therapy. In addition, we also found that combination therapy of Gal-9-ERC with Rapa dramatically improved allograft survival in a synergistic manner, rather than in an antagonistic manner, which would optimize ERC-based cell therapy. Although these results are inspiring and encouraging, further long-term and in-depth studies focusing on evaluations of chronic rejection and vascular lesions are warranted.

## Conclusion

In this study, we showed for the first time that ERCs could express Gal-9 and found this expression was increased by IFN-γ stimulation in a dose-dependent manner. Moreover, we respectively co-cultured Gal-9-ERC with allogenic splenocytes and infused Gal-9-ERC with Rapa to the cardiac allograft recipients. The results demonstrated that Gal-9-ERC-mediated therapy could assist in suppressing allogeneic Th1 and Th17 cell response, inhibiting CD8^+^ T cell proliferation, abrogating B cell activation, decreasing donor-specific antibody production, and promoting Tregs. The excellent immunomodulatory effect was further convinced by the verification for prolongation of allograft survival time and alleviated pathological manifestations. Given together, this study provides a novel idea for supplementing the ERC immunoregulatory mechanism and also offers a promising immunomodulation strategy to be further implemented in the clinical settings.

## Supplementary information

**Additional file 1: **Supplementary Figure 1. Gal-9 Expression in ERC Culture Supernatant. (A) Gal-9 secretion was measured in p2-p5 ERC culture supernatant by ELISA. (B) Gal-9 secretion was measured in IFN-γ-pre-stimulated ERC supernatant. Statistical analysis was performed by one-way analysis of variance (ANOVA), and the post-test was the least significant difference (LSD) test, *n* = 3. * *p* < 0.05 and ** *p* < 0.01. Bar graphs represent mean ± SD. Abbreviation: ERC, endometrial regenerative cell.

**Additional file 2: **Supplementary Figure 2. Gal-9-ERC suppress CD8^+^Tim-3^+^ and CD4^+^Tim3^+^ T cell proliferation in vitro. (A) Representative dot plots of CD8^+^Tim3^+^ T cells. (B) Percentage of CD8^+^Tim-3^+^ T cells. (C) Representative dot plots of CD4^+^Tim-3^+^ T cells. (D) Percentage of CD4^+^Tim-3^+^ T cells. (*n* = 6). Differences among groups were assessed by using one-way analysis of variance (ANOVA). * p < 0.05, ** p < 0.01.

## Data Availability

All data generated or analyzed during this study are included in this published article.

## Abbreviations

ERCs: Endometrial regenerative cells
Gal-9: Galectin-9
MHC: Major histocompatibility complex
CAV: Cardiac allograft vasculopathy
MSCs: Mesenchymal stem cells
CXCR-4: C-X-C chemokine receptor type 4
Tim-3: T cell immunoglobulin mucin-3
Th1: T helper type 1
aGVHD: Acute graft-vs-host disease
iTreg: Induced regulatory T cell
Rapa: Rapamycin
POD: Postoperative day
H&E: Hematoxylin-eosin
MST: Mean survival time
ACR: Acute cellular rejection
DSA: Donor-specific antibody
AMR: Antibody-mediated rejection
DCs: Dendritic cells
APCs: Antigen-presenting cells
