# Correction: Galectin-9 is required for endometrial regenerative cells to induce long-term cardiac allograft survival in mice

**DOI:** 10.1186/s13287-023-03384-7

**Published:** 2023-06-06

**Authors:** Yiming Zhao, Xiang Li, Dingding Yu, Yonghao Hu, Wang Jin, Yafei Qin, Dejun Kong, Hongda Wang, Guangming Li, Alessandro Alessandrini, Hao Wang

**Affiliations:** 1 Department of General Surgery, Tianjin Medical University General Hospital, 154 Anshan Road, Heping District, Tianjin, 300052 China; 2 Tianjin General Surgery Institute, Tianjin Medical University General Hospital, Tianjin, China; 3 Department of Surgery, Center for Transplantation Sciences, Massachusetts General Hospital, Harvard Medical School, Boston, MA USA

**Correction : Stem Cell Research & Therapy (2020) 11:471** **https://doi.org/10.1186/s13287-020-01985-0**

In the original article, the authors noticed that there were inadvertent errors in Figs. 5 and 6.

**Fig. 5:**
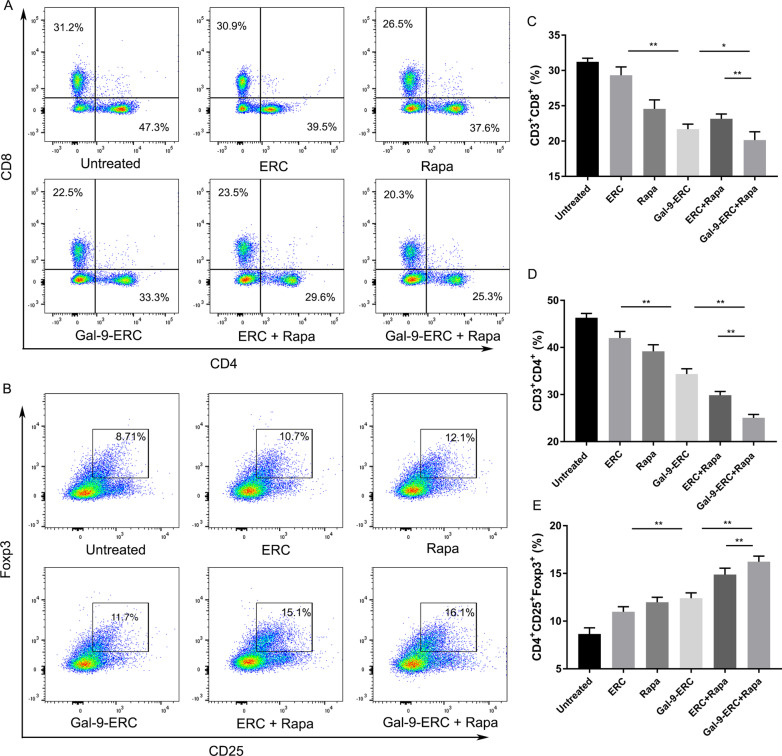
Gal-9-ERC-based therapy inhibited CD4^+^T and CD8^+^T cell response while promoting Treg generation. Immune cells (CD4^+^T and CD8^+^T) activation and proliferation reflect the severity of acute cellular rejection (ACR). Splenocytes from B6 recipients among each group were harvested on POD8 and then stained for flow cytometry analysis. **a** Representative dot plots of CD4^+^T (CD3^+^CD4^+^) and CD8^+^T (CD3^+^CD8^+^) cells. **b** Representative dot plots of Treg (CD4^+^CD25^+^Foxp3^+^) cells. c–e Percentage of CD8^+^ T (CD3^+^ CD8^+^), CD4^+^ T (CD3^+^ CD4^+^) and Treg (CD4^+^CD25^+^Foxp3^+^) cells (n = 6). Differences among groups were assessed by using one-way analysis of variance (ANOVA). *p < 0.05, **p < 0.01. Abbreviations: ERC, endometrial regenerative cell; Gal-9-ERC, Galectin-9 high-expression ERC; POD, postoperative day; Rapa, rapamycin

**Fig. 6:**
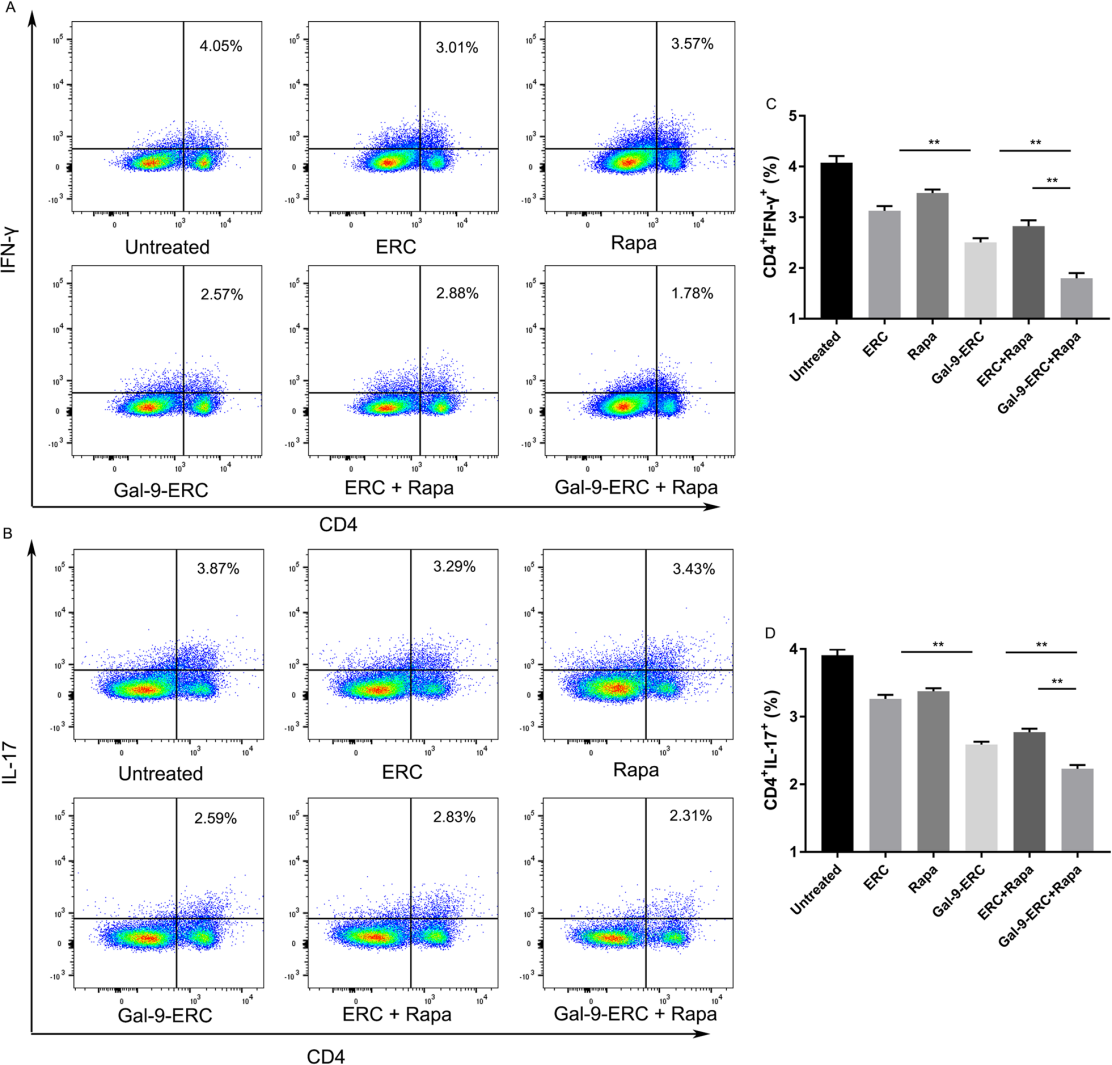
Gal-9-ERC-based therapy modulated T helper cell percentages. As observed in vitro experiment, and transcription factor expression differences in allografts, we would like to further evaluate Gal-9-ERC-based therapy in modulating T helper cells. Splenocytes were harvested on POD8 and stained for FACS. **a**, **b** Representative dot plots of Th1 (CD4^+^IFN-γ^+^) and Th17 (CD4^+^IL-17^+^) cells. **c**, **d** Percentage of Th1 (CD4^+^IFN-γ^+^) and Th17 (CD4^+^IL-17^+^) cells (n = 6). Differences among groups were assessed by using one-way analysis of variance (ANOVA). *p < 0.05, **p < 0.01. Abbreviations: ERC, endometrial regenerative cell; Gal-9-ERC, Galectin-9 high-expression ERC; Rapa, rapamycin

In Fig. 5A: The wrong representative figures in ERC and Rapa groups as published were placed in the process of copying/pasting original images. The authors have now presented the corrected figures and reanalyzed the data below:

In Fig. 6: The original flow cytometry sub-figures in the upper panels (Fig. 6A) were duplicates of those in the low panel (Fig. 6B). During the figure assembly process, the authors forgot to replace the dot plot images after completing the labels in Fig. 6A, which caused this mistake. It has now been replaced with the right sub-figures:

The authors state that these mistakes do not affect the conclusion of the article.

