# Supplementary figures and images for: Galectin-9 is required for endometrial regenerative cells to induce long-term cardiac allograft survival in mice
Source: Stem Cell Res Ther. 2020 Nov 5;11:471. doi: 10.1186/s13287-020-01985-0 (PMC7643467; doi:10.1186/s13287-020-01985-0)

A

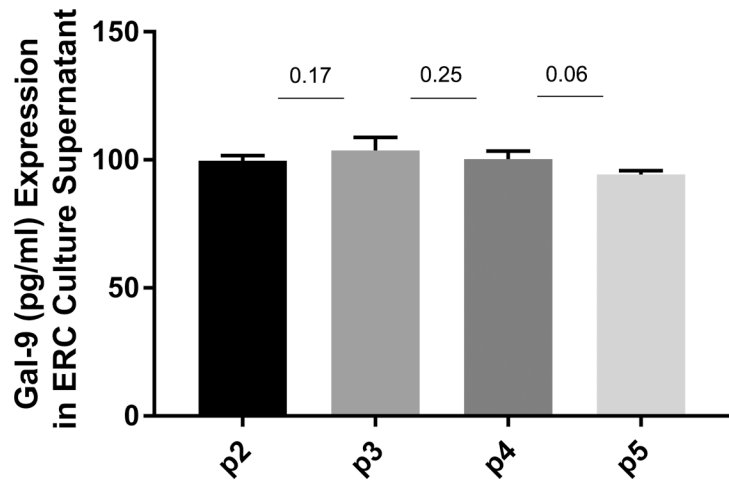

B

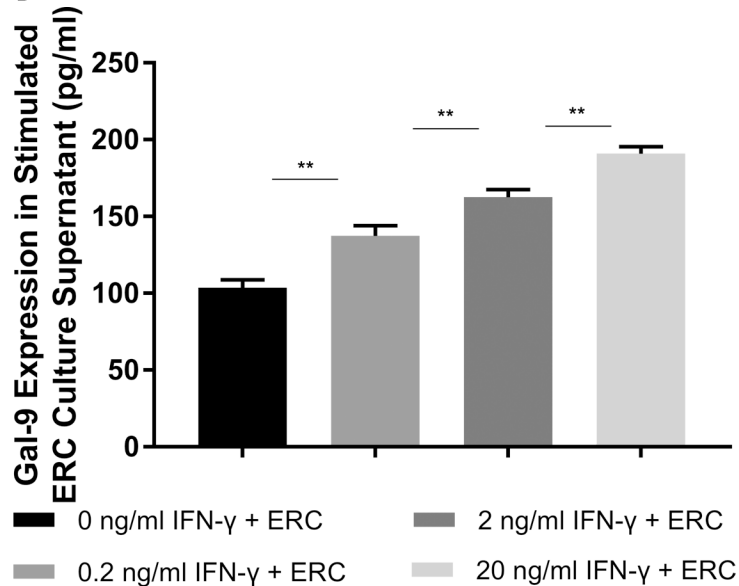

Supplement: Supplementary file 1 — Additional file 1: Supplementary Figure 1. Gal-9 Expression in ERC Culture Supernatant. (A) Gal-9 secretion was measured in p2-p5 ERC culture supernatant by ELISA. (B) Gal-9 secretion was measured in IFN-γ-pre-stimulated ERC supernatant. Statistical analysis was performed by one-way analysis of variance (ANOVA), and the post-test was the least significant difference (LSD) test, n = 3. * p < 0.05 and ** p < 0.01. Bar graphs represent mean ± SD. Abbreviation: ERC, endometrial regenerative cell. [file 13287_2020_1985_MOESM1_ESM.pdf]

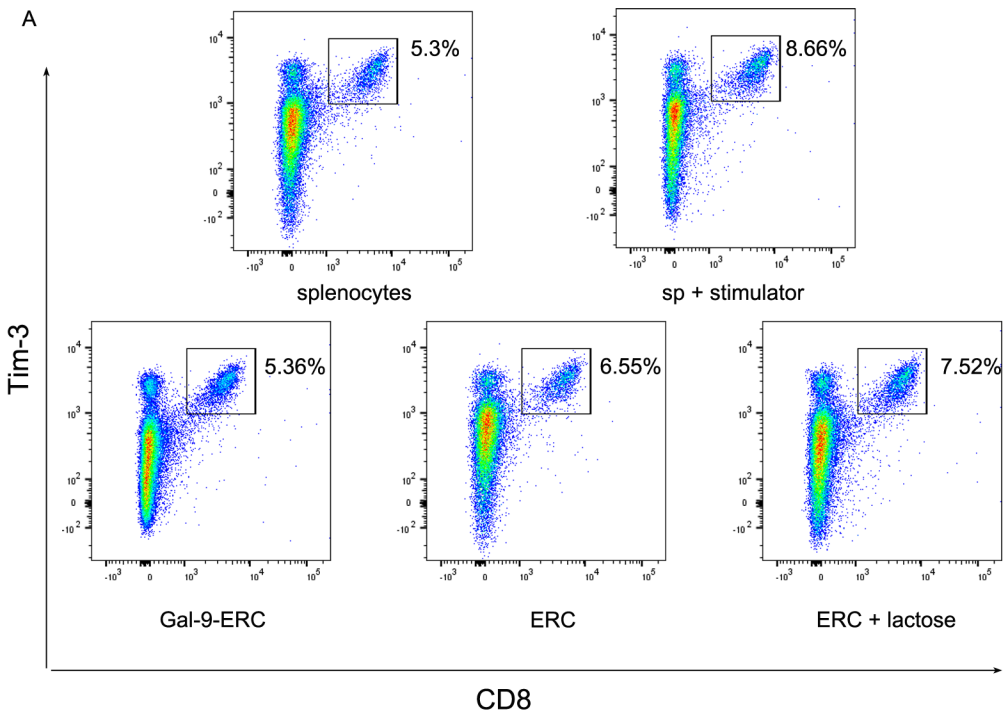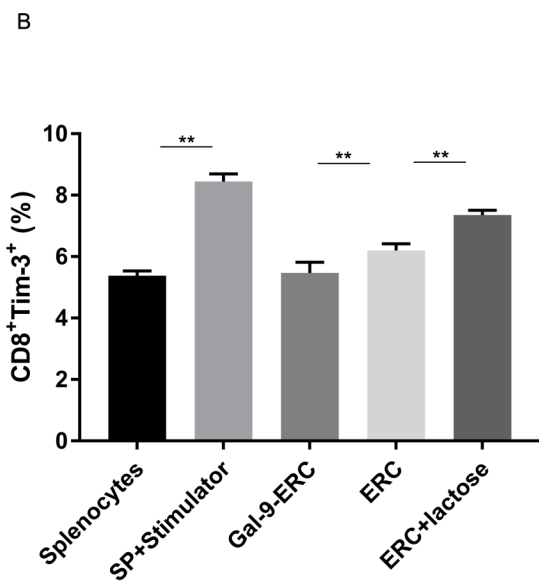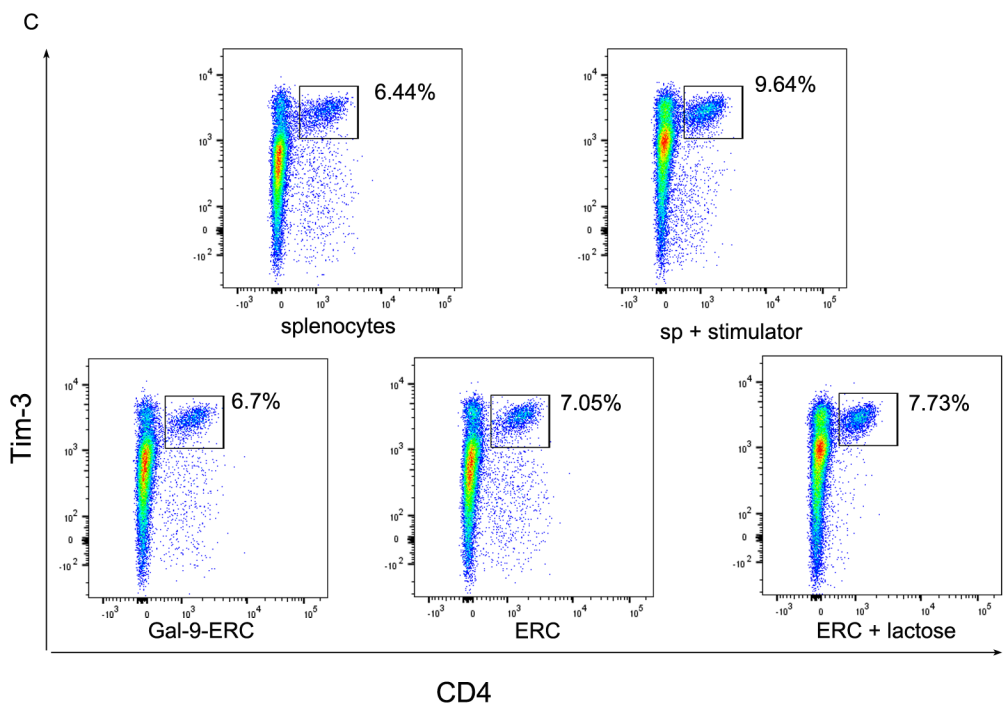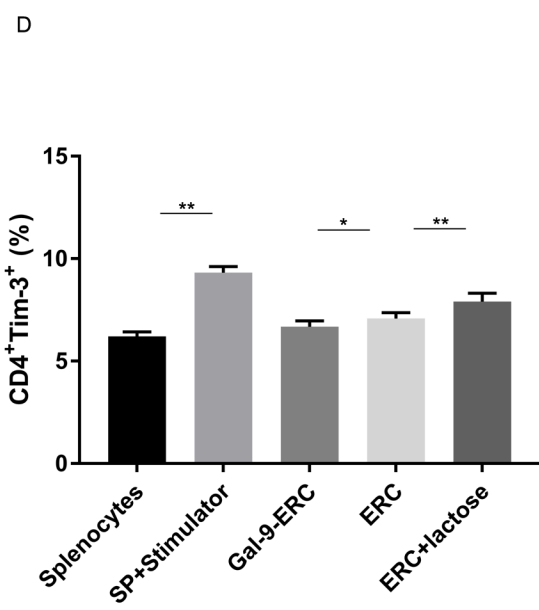

Supplement: Supplementary file 2 — Additional file 2: Supplementary Figure 2. Gal-9-ERC suppress CD8+Tim-3+ and CD4+Tim3+ T cell proliferation in vitro. (A) Representative dot plots of CD8+Tim3+ T cells. (B) Percentage of CD8+Tim-3+ T cells. (C) Representative dot plots of CD4+Tim-3+ T cells. (D) Percentage of CD4+Tim-3+ T cells. (n = 6). Differences among groups were assessed by using one-way analysis of variance (ANOVA). * p < 0.05, ** p < 0.01. [file 13287_2020_1985_MOESM2_ESM.pdf]
